# Association of Sex and Race With Incident Peripheral Artery Disease Among Veterans With Normal Ankle-Brachial Indices

**DOI:** 10.1001/jamanetworkopen.2022.40188

**Published:** 2022-11-03

**Authors:** Aaron W. Aday, Meredith S. Duncan, Olga V. Patterson, Scott L. DuVall, Patrick R. Alba, Charles W. Alcorn, Hilary A. Tindle, Mark A. Creager, Marc P. Bonaca, Scott M. Damrauer, Quinn S. Wells, Adam Behroozian, Joshua A. Beckman, Matthew S. Freiberg

**Affiliations:** 1 Vanderbilt Translational and Clinical Cardiovascular Research Center, Division of Cardiovascular Medicine, Department of Medicine, Vanderbilt University Medical Center, Nashville, Tennessee; 2 Department of Biostatistics, College of Public Health, University of Kentucky, Lexington; 3 VA Informatics and Computing Infrastructure, VA Salt Lake City Health Care System, Salt Lake City, Utah; 4 Department of Internal Medicine, University of Utah School of Medicine, Salt Lake City; 5 University of Pittsburgh School of Public Health, Pittsburgh, Pennsylvania; 6 Department of Medicine, Vanderbilt University Medical Center, Nashville, Tennessee; 7 Heart and Vascular Center, Dartmouth-Hitchcock Medical Center, Geisel School of Medicine at Dartmouth, Lebanon, New Hampshire; 8 Colorado Prevention Center Clinical Research, Division of Cardiovascular Medicine, Department of Medicine, University of Colorado Anschutz Medical Campus, Aurora; 9 Department of Surgery, Perelman School of Medicine, University of Pennsylvania, Philadelphia; 10 Corporal Michael Crescenz VA Medical Center, Philadelphia, Pennsylvania; 11 Now with Division of Cardiovascular Diseases, Scripps Clinic, La Jolla, California; 12 Veterans Affairs Tennessee Valley Healthcare System, Nashville

## Abstract

**Importance:**

Reported risk of incident peripheral artery disease (PAD) by sex and race varies significantly and has not been reported in national cohorts among individuals free of baseline PAD.

**Objective:**

To evaluate the association of sex and race, as well as prevalent cardiovascular risk factors, with limb outcomes in a national cohort of people with normal baseline ankle-brachial indices (ABIs).

**Design, setting, and participants:**

This cohort study was conducted using data from participants in the Veterans Affairs Birth Cohort Study (born 1945-1965), with follow-up data between January 1, 2000, and December 31, 2016. Baseline demographics were collected from 77 041 participants receiving care from the Veterans Health Administration with baseline ABIs of 0.90 to 1.40 and no history of PAD. Data were analyzed from October 2019 through September 2022.

**Exposures:**

Sex, race, diabetes, and smoking status.

**Main Outcomes and Measures:**

Incident PAD, defined as subsequent ABI less than 0.90, surgical or percutaneous revascularization, or nontraumatic amputation.

**Results:**

Of 77 041 participants with normal ABIs (73 822 [95.8%] men; mean [SD] age, 60.2 [5.9] years; 13 080 Black [18.2%] and 54 377 White [75.6%] among 71 911 participants with race and ethnicity data), there were 6692 incident PAD events over a median [IQR] of 3.9 [1.7-6.9] years. Incidence rates were lower for women than men (incidence rates [IRs] per 1000 person-years, 7.4 incidents [95% CI, 6.2-8.8 incidents] vs 19.2 incidents [95% CI, 18.7-19.6 incidents]), with a lower risk of incident PAD (adjusted hazard ratio [aHR], 0.49 [95% CI, 0.41-0.59]). IRs per 1000 person-years of incident PAD were similar for Black and White participants (18.9 incidents [95% CI, 17.9-20.1 incidents] vs 18.8 incidents [95% CI, 18.3-19.4]). Compared with White participants, Black participants had increased risk of total PAD (aHR, 1.09 [95% CI, 1.02-1.16]) and nontraumatic amputation (aHR, 1.20 [95% CI, 1.06-1.36]) but not surgical or percutaneous revascularization (aHR, 1.10 [95% CI, 0.98-1.23]) or subsequent ABI less than 0.90 (aHR, 1.04 [95% CI, 0.95-1.13]). Diabetes (aHR, 1.62 [95% CI, 1.53-1.72]) and smoking (eg, current vs never: aHR, 1.76 [95% CI, 1.64-1.89]) were associated with incident PAD. Incident PAD was rare among individuals without a history of smoking or diabetes (eg, among 632 women: IR per 1000 people-years, 2.1 incidents [95% CI, 1.0-4.5 incidents]) despite an otherwise–high-risk cardiovascular profile (eg, 527 women [83.4%] with hypertension).

**Conclusions and Relevance:**

This study found that the risk of PAD was approximately 50% lower in women than men and less than 10% higher for Black vs White participants, while the risk of nontraumatic amputation was 20% higher among Black compared with White participants.

## Key Points

**Question:**

Are sex and race associated with peripheral artery disease (PAD) incidence?

**Findings:**

In this cohort study of 77 041 veterans with normal baseline ankle-brachial indices and no history of PAD, women were at lower risk of developing PAD than men. While Black and White participants had similar rates of overall PAD, Black participants had increased risk of amputation and White participants had increased risk of surgical or percutaneous revascularization.

**Meaning:**

This study found that the risk of PAD was lower in women than men and the risk of amputation but not revascularization was higher in Black vs White participants.

## Teaser

This cohort study investigates the association of sex and race with incidence of peripheral artery disease among veterans with normal baseline ankle-brachial indices.

## Introduction

Lower-extremity peripheral artery disease (PAD) is common and occurs among 200 million individuals worldwide.^1^ Patients with PAD are at increased risk of cardiac, cerebrovascular, and limb-related morbidity and mortality.^2^ Traditional cardiovascular risk factors, including older age,^3^ smoking,^4,5^ and diabetes,^6,7^ are also associated with increased risk of incident PAD.

In contrast to coronary artery and cerebrovascular disease, data on the association of sex and race with PAD are mixed. Prevalent PAD, defined only by intermittent claudication, was nearly twice as common in men compared with women.^8,9,10^ However, when PAD was defined only by an abnormal ankle-brachial index (ABI),^11^ men had a similar or lower prevalence of PAD compared with women.^9,10,12^ Prior studies^13,14,15,16^ have typically reported increased risks for PAD among Black compared with non-Hispanic White individuals, but these reports were limited by small sample sizes and heterogeneity in PAD end points. Prospective epidemiologic studies with baseline normal and serial ABI measures are uncommon.^17,18^

To address this knowledge gap, we investigated the associations of sex and race with incident PAD in a large, diverse cohort of US veterans, all of whom had normal resting ABIs at baseline. We further examined these associations among individuals with and without diabetes and smoking histories and evaluated subsets of PAD outcomes, including subsequent ABI less than 0.90, surgical or percutaneous revascularization, and nontraumatic amputation.

## Methods

Institutional review boards (IRBs) of the Tennessee Valley Veterans Affairs Health Care System, West Haven VA Medical Center, and Vanderbilt University Medical Center approved this cohort study. The Veterans Affairs Birth Cohort is compliant with the Health Insurance Portability and Accountability Act and has a waiver of consent from the West Haven VA IRB and the Yale University IRB because this was observational research done on deidentified data; the exemption extends to this study. Our study follows the Strengthening the Reporting of Observational Studies in Epidemiology (STROBE) reporting guideline for cohort studies.

### Sample

We derived the study population from participants in the Veterans Affairs Birth Cohort,^19,20^ an observational, prospective cohort that consists of approximately 4.2 million US veterans born between 1945 and 1965 and receiving care at a Veterans Health Administration (VHA) facility. We included electronic health record data obtained from these individuals between January 1, 2000, and December 31, 2016. Participants had a minimum of 2 medical encounters during this time to ensure that they were actively receiving care at a VHA facility.

The study population initially consisted of 255 014 VA Birth Cohort participants who underwent ABI testing within the VHA from January 1, 2000, through December 31, 2016. We excluded participants with any of the following: baseline resting ABI values less than 0.90 (103 002 participants) or greater than 1.40 (5624 participants), resting ABI in only 1 limb (64 029 participants), or prevalent PAD, defined by the presence of VHA, VHA fee-for-service, or Medicare *International Classification of Diseases, Ninth Revision *(*ICD-9*) or *International Statistical Classification of Diseases and Related Health Problems, Tenth Revision *(*ICD-10*), or *Current Procedural Terminology* (*CPT*) codes (eTable 1 in the Supplement) (5318 participants).^21^ Baseline characteristics of individuals excluded from the study are reported in eTable 2 in the Supplement. The final study population consisted of 77 041 participants (eFigure 1 in the Supplement). Baseline was the date of initial normal ABI measurement.

### Independent Variables

Primary exposures were sex and race collected from VHA administrative data. Sex, race, and ethnicity were obtained from demographic information included in the Veterans Administration Corporate Data Warehouse, where data from VHA electronic health records are stored. This information was collected administratively by the VA at point of service. Prior studies^22,23^ have shown a high concordance between self-identified race and ethnicity with that reported in the Corporate Data Warehouse. Race and ethnicity were categorized as Black, White, or other, which included individuals identified as American Indian or Alaska Native, Asian, and Native Hawaiian or other Pacific Islander and those with Hispanic ethnicity. Given the small sample sizes of groups in the other category, our analyses focused on Black and White individuals. Additional exposures included diabetes and smoking history. The former was a validated metric that included administrative codes, medications, and laboratory values^24^; the latter was categorized as current, past, or never based on data for validated health risk factors within the Veterans Affairs electronic health record.^25^

### Dependent Variables

We defined the primary outcome, incident PAD, as a composite of the following: subsequent resting ABI less than 0.90 in either limb, surgical or percutaneous lower limb artery revascularization, and nontraumatic lower-extremity amputation. We extracted ABI data directly from the electronic health record using a validated, VHA-developed natural language–processing tool, which permits collection of ABI results from multiple locations within the electronic health record.^26^ We defined lower-extremity revascularization and amputation as the presence of 1 or more inpatient or 2 or more outpatient VHA, VHA fee-for-service, or Medicare *ICD-9* or *ICD-10* codes or 1 or more *CPT* codes (eTable 1 in the Supplement). Participants did not meet our criteria for PAD-associated amputation if trauma or other unrelated administrative codes (eTable 3 in the Supplement) were also present within 30 days of an otherwise-qualifying PAD event. Amputation procedure codes within the VHA have been found to be valid with a 100% positive predictive value for a single amputation procedure code.^27^ Additionally, using patient health records, we manually adjudicated a random sample of 50 incident amputations and confirmed all 50 as nontraumatic lower-extremity amputations. Exploratory analyses examined each component of the composite PAD variable separately. Participants were followed up from their baseline date to a PAD event, death, or the censoring date (December 31, 2016).

### Covariates

Included covariates were age, systolic and diastolic blood pressure, antihypertensive medication use, body mass index (BMI; calculated as weight in kilograms divided by height in meters squared), estimated glomerular filtration rate (eGFR), high-density lipoprotein cholesterol (HDL-C) level, low-density lipoprotein cholesterol (LDL-C) level, triglyceride level, statin use, and prevalent cardiovascular disease (CVD) (ie, myocardial infarction, stroke, coronary heart disease, or congestive heart failure). These variables were chosen a priori based on known associations with incident atherosclerotic disease, including PAD.^2^ Covariates were recorded using outpatient and clinical laboratory data closest to the baseline date described previously and based on our prior work.^24,28^

### Missing Data

We accounted for missing data using multiple imputation via chained equation techniques to produce 10 complete data sets. The number of missing values per variable is displayed in the footnote of Table 1. All variables had fewer than 10% missing values, with the exception of smoking status, which was missing among 14 458 individuals (18.8%). All variables reported in Table 1, along with time to PAD and an indicator of incident PAD, were included as factors in multiple imputation models. We imputed continuous variables using predictive mean matching to produce biologically plausible values,^29^ while we imputed categorical variables using the discriminant function with a noninformative Jeffrey prior.^30^ We combined results over imputations according to Rubin rules.^31^

**Table 1.** Baseline Characteristics of Study Participants

Characteristic	Participants, No. (%) (N = 77 041)				
	Sex		Race and ethnicity		
	Men (n = 73 822)^a^	Women (n = 3219)^b^	Black (n = 13 080)^c^	White (n = 54 377)^d^	Other (n = 4454)^e^^,^^f^
Age, mean (SD), y	60.2 (5.9)	56.1 (6.3)	58.6 (6.1)	60.5 (5.8)	59.7 (5.9)
Sex					
Men	NA	NA	12 200 (93.3)	52 419 (96.4)	4297 (96.5)
Women	NA	NA	880 (6.7)	1958 (3.6)	157 (3.5)
Race and ethnicity					
Black	12 200 (17.7)	880 (29.4)	NA	NA	NA
White	52 419 (76.1)	1958 (65.4)	NA	NA	NA
Other^f^	4297 (6.2)	157 (5.2)	NA	NA	NA
Smoking status					
Current	28 150 (49.2)	1113 (42.0)	5241 (48.0)	20 933 (47.3)	1242 (35.8)
Former	17 806 (27.5)	542 (20.4)	2705 (24.8)	13 533 (30.6)	1029 (29.7)
Never	13 980 (23.3)	992 (37.5)	2981 (27.3)	9724 (22.0)	1196 (34.5)
Diabetes	40 583 (55.0)	1199 (37.3)	7707 (58.9)	28 733 (52.8)	2820 (63.3)
Prevalent CVD	32 862 (44.5)	864 (26.8)	5031 (38.5)	24 791 (45.6)	1864 (41.9)
ABI, median (IQR)					
Left	1.1 (1.0-1.2)	1.1 (1.0-1.2)	1.1 (1.0-1.2)	1.1 (1.0-1.2)	1.1 (1.0-1.2)
Right	1.1 (1.0-1.2)	1.1 (1.0-1.2)	1.1 (1.0-1.2)	1.1 (1.0-1.2)	1.1 (1.0-1.2)
Blood pressure, mean (SD), mm Hg					
SBP	129.6 (13.1)	127.5 (13.2)	131.1 (13.9)	129.1 (12.8)	129.7 (12.9)
DBP	76.4 (8.5)	75.4 (8.6)	78.4 (8.7)	75.8 (8.3)	76.1 (8.5)
Antihypertensive therapy	70 670 (95.7)	2896 (90.0)	12 585 (96.2)	52 158 (95.9)	4306 (96.7)
BMI, mean (SD)	31.7 (7.1)	31.4 (7.3)	30.9 (7.0)	31.9 (7.1)	31.9 (6.7)
Cholesterol, median (IQR), mg/dL					
Total	167 (142-197)	191 (165-222)	169 (143-198)	168 (142-197)	168 (141-199)
LDL	94 (73-119)	109 (87-136)	96 (74-121)	94 (73-120)	94 (72-121)
HDL	39 (33-48)	50 (42-62)	44 (36-54)	39 (33-47)	40 (33-48)
Triglycerides	139 (94-209)	126 (85-190)	110 (77-164)	145 (99-217)	144 (99-211)
Statin therapy	60 123 (81.4)	2278 (70.8)	10 102 (77.2)	44 959 (82.7)	3715 (83.4)
eGFR, mean (SD), mL/min/1.73m^2^	86.8 (27.0)	84.4 (24.2)	90.0 (30.3)	85.9 (25.4)	86.2 (28.7)

Abbreviations: ABI, ankle-brachial index; BMI, body mass index (calculated as weight in kilograms divided by height in meters squared); CVD, cardiovascular disease; DBP, diastolic blood pressure; eGFR, estimated glomerular filtration rate; HDL, high-density lipoprotein; LDL, low-density lipoprotein; NA, not applicable; SBP, systolic blood pressure.

SI conversion factors: To convert HDL, LDL, and total cholesterol to millimoles per liter, multiply by 0.0259; triglycerides to millimoles per liter, multiply by 0.0113.

^a^ Number missing: 4906 men for race and ethnicity, 13 886 men for smoking status, 1785 men for SBP, 1785 men for DBP, 939 men for BMI, 2254 men for total cholesterol, 2786 men for LDL cholesterol, 2585 men for HDL cholesterol, 2664 men for triglycerides, and 916 men for eGFR.

^b^ Number missing: 224 women for race and ethnicity, 572 women for smoking status, 344 women for SBP, 344 women for DBP, 54 women for BMI, 108 women for total cholesterol, 134 women for LDL cholesterol, 127 women for HDL cholesterol, 127 women for triglycerides, and 70 women for eGFR.

^c^ Number missing: 2153 participants for smoking status, 389 participants for SBP, 389 participants for DBP, 124 participants for BMI, 351 participants for total cholesterol, 483 participants for LDL cholesterol, 438 participants for HDL cholesterol, 434 participants for triglycerides, and 110 participants for eGFR.

^d^ Number missing: 10 169 participants for smoking status, 1135 participants for SBP, 1135 participants for DBP, 501 participants for BMI, 1432 participants for total cholesterol, 1730 participants for LDL cholesterol, 1603 participants for HDL cholesterol, 1678 participants for triglycerides, and 579 participants for eGFR.

^e^ Number missing: 987 participants for smoking status, 122 participants for SBP, 122 participants for DBP, 56 participants for BMI, 114 participants for total cholesterol, 141 participants for LDL cholesterol, 130 participants for HDL cholesterol, 136 participants for triglycerides, and 46 participants for eGFR.

^f^ Other race and ethnicity included individuals identified as American Indian or Alaska Native, Asian, and Native Hawaiian or other Pacific Islander, as well as those with Hispanic ethnicity.

### Statistical Analysis

We summarized continuous data as mean (SD) or median (IQR) depending on the normality of their distributions, and we reported categorical data as counts and percentages. We estimated unadjusted PAD incidence rates by Poisson regression with an offset equal to the natural logarithm of follow-up time. We stratified cumulative incidence curves by sex, race, and ethnicity and analyzed them using a log-rank test. After confirming that the proportional hazards assumption was not violated via examination of log-log plots, we used Cox proportional hazards regression to estimate age-adjusted and multivariable-adjusted cause-specific hazard ratios (HRs) for PAD risk by sex, race and ethnicity, diabetes, and smoking status.^32^ Multivariable-adjusted models included age, systolic and diastolic blood pressure, antihypertensive medication use, BMI, eGFR, HDL-C level, LDL-C level, statin use, and prevalent CVD as covariates. In sensitivity analyses, we limited to individuals without diabetes, those who had never smoked, and their intersection to assess PAD risk in those without these risk factors. Additional sensitivity analyses examined the number of individuals who died during follow-up to assess the possibility that our findings were associated with competing risk of death. Secondary analyses examined components of incident PAD separately. All HRs reported in text are multivariable adjusted. We considered a 2-sided *P* value < .05 statistically significant for log-rank tests. We performed multiple imputation in R statistical software version 3.6.2 (R Project for Statistical Computing), and we used SAS statistical software version 9.4 (SAS Institute) for all other analyses. Data were analyzed from October 2019 through September 2022.

## Results

There were 77 041 total participants (73 822 [95.8%] men and 3219 [4.2] women; mean [SD] age, 60.2 [5.9] years; 13 080 Black [18.2%], 54 377 White [75.6%], and 4454 individuals with other race or ethnicity [6.2%] among 71 911 participants with race and ethnicity data). Women had a lower prevalence of smoking (1113 women [42.0%] vs 28 150 men [49.2%]), diabetes (1199 women [37.3%] vs 40 583 men [55.0%]), CVD (864 women [26.8%] vs 32 862 [44.5%]), and statin therapy (2278 women [70.8%] vs 60 123 men [81.4%]) compared with men (Table 1). Black participants, compared with White participants, had a higher prevalence of never smoking (2981 of 10 927 participants [27.3%] vs 9724 of 44 208 participants [22.0%] with race and smoking data) and diabetes (7707 of 13 080 participants [58.9%] vs 28 733 of 54 377 participants [52.8%] with race and diabetes data). In contrast, Black participants had a lower prevalence of CVD (5031 of 13 080 participants [38.5%] vs 24 791 of 54 377 participants [45.6%] with race and CVD data) and statin therapy (10 102 of 13 080 participants [77.2%] vs 44 959 of 54 377 participants [82.7%] with race and statin data) compared with White participants. The median (IQR) baseline ABI among all groups in each limb was 1.1 (1.0-1.2). There were 6692 incident PAD events over a median (IQR) follow-up period of 3.9 (1.7-6.9) years.

Cumulative incidence of PAD was significantly lower in women than men (Figure, A) but similar across race and ethnicity categories (Figure, B). Individuals with diabetes and those who were current smokers had greater cumulative incidence of PAD than those without diabetes (eFigure 2 in the Supplement) and former or never smokers (eFigure 3 in the Supplement).

**Figure.**
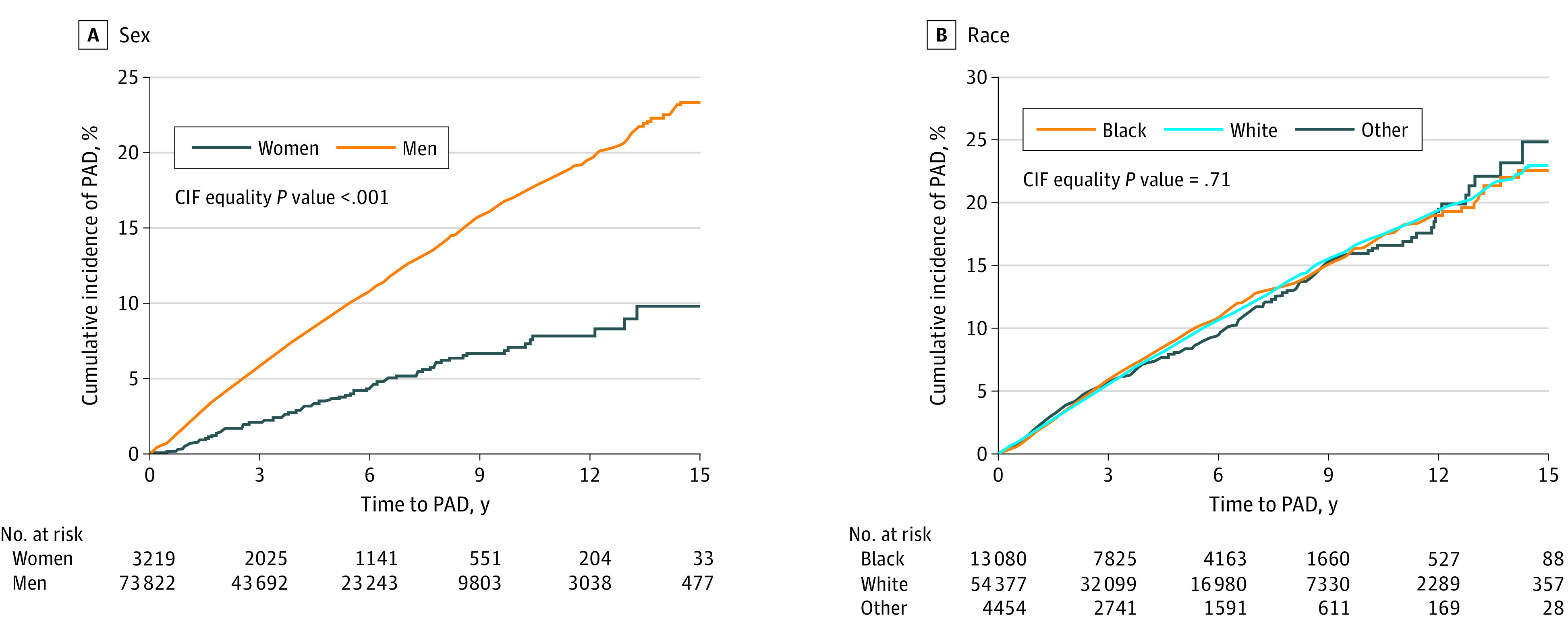
Cumulative Incidence of Peripheral Artery Disease (PAD) Incident PAD was defined by ankle-brachial index less than 0.90, nontraumatic amputation, or surgical or percutaneous lower-extremity arterial revascularization. CIF indicates cumulative incidence function.

Unadjusted PAD incidence rates were lower among women compared with men (incidence rates [IRs] per 1000 person-years, 7.4 incidents [95% CI, 6.2-8.8 incidents] vs 19.2 incidents [95% CI, 18.7-19.6 incidents]) (Table 2). IRs per 1000 person-years were similar for Black and White participants (18.9 incidents [95% CI, 17.9-20.1 incidents] vs 18.8 incidents [95% CI, 18.3-19.4]), with no difference in time to incident PAD (eFigure 4 in the Supplement). Individuals with diabetes had higher IRs per 1000 person-years than those without diabetes (22.6 incidents [95% CI, 21.9-23.2 incidents] vs 13.5 incidents [95% CI, 13.0-14.1 incidents]). Similarly, there was an increase in IRs per 1000 person-years across participants who were never, former, and current smokers (12.3 incidents [95% CI, 11.5-13.2 incidents], 16.7 incidents [95% CI, 15.8-17.6 incidents], and 23.4 incidents [95% CI, 22.6-24.2 incidents], respectively).

**Table 2.** Association of Participant Characteristics With Incident PAD

Characteristic	PAD events/No. of participants	IR per 1000 person-y (95% CI)	aHR (95% CI)	
			Age	Multivariable^a^
Sex				
Male	6571/73 822	19.2 (18.7-19.6)	1 [Reference]	1 [Reference]
Female	121/3219	7.4 (6.2-8.8)	0.40 (0.33-0.47)	0.49 (0.41-0.59)
Race and ethnicity^b^				
Black	1153/13 080	18.9 (17.9-20.1)	1.03 (0.97-1.10)	1.09 (1.02-1.16)
White	4756/54 377	18.8 (18.3-19.4)	1 [Reference]	1 [Reference]
Other^c^	388/4454	18.0 (16.3-19.9)	0.96 (0.86-1.06)	0.98 (0.88-1.09)
Unknown	395/5130	16.2 (14.7-17.9)	NA	NA
Diabetes				
Absent	2129/35 259	13.5 (13.0-14.1)	1 [Reference]	1 [Reference]
Present	4563/41 782	22.6 (21.9-23.2)	1.67 (1.58-1.75)	1.62 (1.53-1.72)
Smoking status^b^				
Never	877/14 972	12.3 (11.5-13.2)	1 [Reference]	1 [Reference]
Former	1359/18 348	16.7 (15.8-17.6)	1.31 (1.21-1.42)	1.23 (1.14-1.33)
Current	3273/29 263	23.4 (22.6-24.2)	1.90 (1.77-2.04)	1.76 (1.64-1.89)
Unknown	1183/14 458	17.7 (16.7-18.7)	NA	NA

Abbreviations: aHR, adjusted hazard ratio; IR, incidence rate; NA, not applicable; PAD, peripheral artery disease.

^a^ Model includes the following independent variables: sex, race and ethnicity, diabetes, smoking status, age, systolic blood pressure, diastolic blood pressure, antihypertensive use, body mass index (calculated as weight in kilograms divided by height in meters squared), estimated glomerular filtration rate, high-density lipoprotein cholesterol level, low-density lipoprotein cholesterol level, triglyceride level, statin use, and prevalent cardiovascular disease. Missing data were multiply imputed using predictive mean matching.

^b^ IRs were calculated using observed (ie, nonimputed) data; missing values were imputed for calculating HRs.

^c^ Other race and ethnicity included individuals identified as American Indian or Alaska Native, Asian, and Native Hawaiian or other Pacific Islander, as well as those with Hispanic ethnicity.

The risk of incident PAD was 51% lower among women than men (adjusted HR [aHR], 0.49 [95% CI, 0.41-0.59]) (Table 2). In contrast, Black participants had a 9% increased risk of PAD compared with White participants (aHR, 1.09 [95% CI, 1.02-1.16]). The presence of diabetes was associated with increased risk of PAD (aHR, 1.62 [95% CI, 1.53-1.72]). Current smokers had an increased risk of incident PAD compared with those who had never smoked (aHR, 1.76 [95% CI, 1.64-1.89]).

Secondary analyses examining sex and race and ethnicity and the risk of incident PAD in individuals without diabetes had similar results to the analysis of the full cohort (eTable 4 in the Supplement). In similar analyses among individuals who had never smoked, women had a lower risk of PAD than men (aHR, 0.51 [95% CI, 0.34-0.77]) while Black participants did not have an increased risk of PAD compared with White participants (aHR, 1.18 [95% CI, 0.99-1.40]) (eTable 5 in the Supplement). In analyses of participants who were never smokers and did not have diabetes, incident PAD was rare, particularly among 632 women (IR per 1000 people-years, 2.1 incidents [95% CI, 1.0-4.5 incidents]) (eTable 6 in the Supplement). Moreover, this low PAD event rate occurred in a population with high prevalence of CVD (92 women [14.6%]), hypertension (eg, 527 women [83.4%] with antihypertensive therapy), and statin use (340 women [53.8%]) (eTable 7 in the Supplement). In analyses exploring competing risk of death, rates of death remained lower among women vs men, individuals without diabetes, and those who were never and former smokers compared with current smokers (eTable 8 in the Supplement). Proportions of participants who died were similar across race and ethnicity groups.

In secondary analyses, we observed similar findings when incident PAD was examined by its separate components: lower-extremity revascularization, amputation, and subsequent nonnormal ABI as separate outcomes. Women were significantly less likely than men to undergo lower-extremity arterial revascularization (aHR, 0.47 [95% CI, 0.34-0.65]) (Table 3). Black participants had a similar likelihood of undergoing revascularization (aHR, 1.10 [95% CI, 0.98-1.23]) and time to revascularization (eFigure 4 in the Supplement) compared with White participants.

**Table 3.** Association of Participant Characteristics With Lower-Extremity Revascularization

Characteristic	Revascularization events/No. of participants	IR per 1000 person-y (95% CI)	aHR (95% CI)	
			Age	Multivariable^a^
Sex				
Male	2029/73 822	5.6 (5.4-5.9)	1 [Reference]	1 [Reference]
Female	38/3219	2.3 (1.7-3.1)	0.39 (0.29-0.54)	0.47 (0.34-0.65)
Race and ethnicity^b^				
Black	363/13 080	5.7 (5.1-6.3)	1.05 (0.94-1.18)	1.10 (0.98-1.23)
White	1445/54 377	5.5 (5.2-5.7)	1 [Reference]	1 [Reference]
Other^c^	126/4454	5.6 (4.7-6.7)	1.02 (0.85-1.22)	1.07 (0.89-1.29)
Unknown	133/5130	5.3 (4.4-6.3)	NA	NA
Diabetes				
Absent	736/35 259	4.5 (4.2-4.9)	1 [Reference]	1 [Reference]
Present	1331/41 782	6.2 (5.9-6.6)	1.36 (1.24-1.49)	1.39 (1.26-1.54)
Smoking status^b^				
Never	240/14 972	3.3 (2.9-3.7)	1 [Reference]	1 [Reference]
Former	375/18 348	4.4 (4.0-4.9)	1.32 (1.13-1.54)	1.25 (1.07-1.46)
Current	1069/29 263	7.2 (6.8-7.6)	2.13 (1.86-2.44)	1.88 (1.63-2.16)
Unknown	383/14 458	5.5 (5.0-6.1)	NA	NA

Abbreviations: aHR, adjusted hazard ratio; IR, incidence rate; NA, not applicable.

^a^ Model includes the following independent variables: sex, race and ethnicity, diabetes, smoking status, age, systolic blood pressure, diastolic blood pressure, antihypertensive use, body mass index (calculated as weight in kilograms divided by height in meters squared), estimated glomerular filtration rate, high-density lipoprotein cholesterol level, low-density lipoprotein cholesterol level, triglyceride level, statin use, and prevalent cardiovascular disease. Missing data were multiply imputed using predictive mean matching.

^b^ IRs were calculated using observed (ie, nonimputed) data; missing values were imputed for calculating HRs.

^c^ Other race and ethnicity included individuals identified as American Indian or Alaska Native, Asian, and Native Hawaiian or other Pacific Islander, as well as those with Hispanic ethnicity.

Women were less likely to undergo nontraumatic lower-extremity amputation than men (aHR, 0.29 [95% CI, 0.18-0.45]) (Table 4). Black participants were more likely to undergo amputation compared with White participants (aHR, 1.20 [95% CI, 1.06-1.36]) but had no difference in time to amputation (eFigure 4 in the Supplement).

**Table 4.** Association of Participant Characteristics With Nontraumatic Lower-Extremity Amputation

Characteristic	Amputation events/No. of participants	IR per 1000 person-y (95% CI)	aHR (95% CI)	
			Age	Multivariable^a^
Sex				
Male	1706/73 822	4.7 (4.5-4.9)	1 [Reference]	1 [Reference]
Female	20/3219	1.2 (0.8-1.8)	0.22 (0.14-0.35)	0.29 (0.18-0.45)
Race and ethnicity^b^				
Black	362/13 080	5.7 (5.1-6.3)	1.32 (1.17-1.48)	1.20 (1.06-1.36)
White	1123/54 377	4.2 (4.0-4.5)	1 [Reference]	1 [Reference]
Other^c^	142/4454	6.3 (5.4-7.4)	1.47 (1.23-1.75)	1.26 (1.06-1.50)
Unknown	99/5130	3.9 (3.2-4.7)	NA	NA
Diabetes				
Absent	171/35 259	1.0 (0.9-1.2)	1 [Reference]	1 [Reference]
Present	1555/41 782	7.3 (6.9-7.6)	7.20 (6.14-8.43)	6.59 (5.58-7.80)
Smoking status^b^				
Never	360/14 972	4.9 (4.5-5.5)	1 [Reference]	1 [Reference]
Former	375/18 348	4.4 (4.0-4.9)	0.97 (0.84-1.12)	0.96 (0.83-1.11)
Current	643/29 263	4.3 (4.0-4.6)	0.92 (0.81-1.05)	1.06 (0.93-1.21)
Unknown	348/14 458	5.0 (4.5-5.5)	NA	NA

Abbreviations: aHR, adjusted hazard ratio; IR, incidence rate; NA, not applicable.

^a^ Model includes the following independent variables: sex, race and ethnicity, diabetes, smoking status, age, systolic blood pressure, diastolic blood pressure, antihypertensive use, body mass index (calculated as weight in kilograms divided by height in meters squared), estimated glomerular filtration rate, high-density lipoprotein cholesterol level, low-density lipoprotein cholesterol level, triglyceride level, statin use, and prevalent cardiovascular disease. Missing data were multiply imputed using predictive mean matching.

^b^ IRs were calculated using observed (ie, nonimputed) data; missing values were imputed for calculating HRs.

^c^ Other race and ethnicity included individuals identified as American Indian or Alaska Native, Asian, and Native Hawaiian or other Pacific Islander, as well as those with Hispanic ethnicity.

For subsequent ABI less than 0.90, results were similar to those for total PAD (eTable 9 in the Supplement), with the exception that Black and White participants had similar risks of incident nonnormal ABIs (aHR, 1.04 [95% CI, 0.95-1.13]). Additionally, there was no difference in time to subsequent ABI less than 0.90 between Black and White participants (eFigure 4 in the Supplement).

## Discussion

In this cohort study of a large, diverse group of veterans with normal baseline ABI measures and no history of prevalent PAD, we observed no difference in the risk of lower-extremity revascularization or an incident ABI less than 0.90 between Black and White participants. In contrast, there were substantially and significantly lower rates of PAD among women compared with men and lower rates and risk of amputations among White compared with Black participants. Among participants who were diabetic or current smokers, the risk of incident PAD was high; however, among those who were never smokers and did not have diabetes, incident PAD was rare even though these participants had a high prevalence of CVD, hypertension, and statin use.

Our findings suggest that a more nuanced, contemporary reevaluation of PAD epidemiology may be needed for 2 reasons: first, despite existing public health messaging,^33^ Black participants were not at a uniformly higher risk of PAD compared with White participants, although they had higher rates and risk of amputation. Second, in the absence of diabetes and smoking, participants had a low risk of PAD despite a high prevalence of CVD, antihypertensive use, and lipid-lowering therapy. The former finding challenges the notion that Black individuals are inherently predisposed to a higher risk of PAD, and the latter finding challenges the notion that PAD is simply another manifestation of systemic atherosclerosis. While it is striking that participants with high cardiovascular risk and no diabetes or smoking history had low rates of PAD, we cannot exclude the possibility that high-quality preventive care and genetic predisposition were associated with this outcome. Further investigation of these findings may inform screening strategies, preventive therapy, and improved understanding of the biologic mechanisms driving PAD development.

While prior studies^3,13,14,34^ found a greater prevalence of PAD among Black compared with non-Hispanic White individuals, prospective studies investigating an association between Black race and increased risk of PAD are limited.^6^ In contrast, we examined more than 70 000 participants, including more than 13 000 Black participants, for incident events, and all participants were free of prevalent PAD based on administrative data and normal ABI examinations at baseline. Thus, differences between our study and prior work may be explained by study design and power. Alternatively, the lack of difference in incident PAD by race within the VHA may also be associated with the receipt of care in a unified, national health care system that recognizes the adverse consequences of PAD among its population.^35,36^ However, Black participants had increased rates and risk of nontraumatic lower-extremity amputation compared with White participants even after adjustment for vascular risk factors, which is consistent with findings from a prior study.^37^ The underlying reasons for this disparity in rates of amputation by race are unclear. Importantly, although prior reports suggested that Black patients may present later in the course of PAD, we did not observe differences in time to any PAD event by race. Whether other patient-, clinician-, or system-level factors contribute to this disparity is not clear, but this warrants further investigation within and outside the VHA.

In our study, rates and risk of incident PAD, revascularization, and amputation were markedly lower among women compared with men. While acknowledging that our VA population was overwhelmingly men, this study represents 1 of the largest studies to date of incident PAD among a female population that includes Black women. Our results are also consistent with those among White men and women from the Framingham Heart Study cohorts^10^ but differ from those of some cohorts reporting no difference or modest differences in prevalence of intermittent claudication.^12,38^ Differences between our results and others may be explained by study design, types of PAD outcomes, age at time of entry, or the incorporation of ABI-based diagnostic imaging into PAD definitions. Our study and the Framingham Heart Study cohorts^10^ reported similarly higher rates of PAD among men compared with women, suggesting that our results were not simply a phenomenon among veterans. In both cohorts, risk remained higher for men than women after adjusting for differences in baseline vascular risk factors, suggesting that differences in PAD by sex were not associated with differences in vascular risk factor profiles. Thus, future studies are needed to elucidate the reason or reasons for significantly lower rates of PAD among women.

### Limitations

This study has several limitations. First, our veteran population was overwhelmingly men, and thus our results may not be generalizable to women. However, as mentioned previously, our cohort represents 1 of the largest in which incident PAD was examined among women and that also included Black women. Second, our VA study population included participants with a high burden of cardiovascular risk factors, and thus our results may not be generalizable to a non-VA population. Additionally, some exposures, such as posttraumatic stress disorder, which are more prevalent among veterans than some other populations, may also be associated with PAD risk.^39^ Third, our screening ABI examinations were performed as part of clinical care. Nevertheless, each participant had a normal ABI and thus would not be considered to have prevalent PAD based on current standards of care or research protocols. Fourth, our study did not have detailed data on baseline symptoms or angiographic details of disease. However, our study did use a novel, validated natural language–processing tool to permit extraction of ABI data, which is lacking from other large administrative data sources. Fifth, as with any observational study, there is the possibility of residual confounding.

## Conclusions

In this cohort study of more than 70 000 veterans, including more than 13 000 Black participants and more than 3200 women, we observed no difference in rates and risk of lower-extremity revascularization or incident PAD by an ABI less than 0.90 among Black vs White participants. We observed lower rates of incident PAD among women compared with men and lower rates of amputation among White compared with Black participants. While PAD risk was high among individuals with diabetes and those who smoked, it was rare among participants who were never smokers and did not have diabetes, including those with high cardiovascular risk. Future studies should investigate the reasons for the increased risk of amputation among Black patients, decreased risk of PAD among women, and potential unique mechanisms associated with risk of PAD.

## Notes

Supplement. **eTable 1. **Diagnostic and Procedural Codes for Peripheral Artery Disease **eTable 2.** Baseline Characteristics of Individuals Excluded From the Study **eTable 3. **Procedural Codes for Lower-Extremity Amputation Due to Trauma or Non–Peripheral Artery Disease Indications **eTable 4. **Association of Sex, Race, and Ethnicity With Peripheral Artery Disease Among Individuals Without Diabetes **eTable 5. **Association of Sex, Race, and Ethnicity With Peripheral Artery Disease Among Individual Who Never Smoked **eTable 6. **Association of Sex, Race, and Ethnicity With Peripheral Artery Disease Among Individuals Without Diabetes Who Never Smoked **eTable 7. **Baseline Characteristics of Individuals Without Diabetes Who Never Smoked **eTable 8. **Deaths During Follow-up by Sex, Race and Ethnicity, Diabetes, and Smoking **eTable 9. **Association of Sex, Race and Ethnicity, Diabetes, and Smoking With Subsequent Ankle-Brachial Index Less Than 0.90 **eFigure 1. **Sample Derivation **eFigure 2. **Cumulative Incidence of Peripheral Artery Disease by History of Diabetes **eFigure 3. **Cumulative Incidence of Peripheral Artery Disease by Smoking History **eFigure 4. **Distribution of Time to Incident Peripheral Artery Disease by Race

## References

[1] Fowkes FGR, Rudan D, Rudan I, . Comparison of global estimates of prevalence and risk factors for peripheral artery disease in 2000 and 2010: a systematic review and analysis. Lancet. 2013;382(9901):1329-1340. doi:10.1016/S0140-6736(13)61249-0 23915883

[2] Aday AW, Matsushita K. Epidemiology of peripheral artery disease and polyvascular disease. Circ Res. 2021;128(12):1818-1832. doi:10.1161/CIRCRESAHA.121.318535 34110907 PMC8202714

[3] Allison MA, Ho E, Denenberg JO, . Ethnic-specific prevalence of peripheral arterial disease in the United States. Am J Prev Med. 2007;32(4):328-333. doi:10.1016/j.amepre.2006.12.010 17383564

[4] Ding N, Sang Y, Chen J, . Cigarette smoking, smoking cessation, and long-term risk of 3 major atherosclerotic diseases. J Am Coll Cardiol. 2019;74(4):498-507. doi:10.1016/j.jacc.2019.05.049 31345423 PMC6662625

[5] Conen D, Everett BM, Kurth T, . Smoking, smoking cessation, [corrected] and risk for symptomatic peripheral artery disease in women: a cohort study. Ann Intern Med. 2011;154(11):719-726. doi:10.7326/0003-4819-154-11-201106070-00003 21646555 PMC3111942

[6] Matsushita K, Sang Y, Ning H, . Lifetime risk of lower-extremity peripheral artery disease defined by ankle-brachial index in the United States. J Am Heart Assoc. 2019;8(18):e012177. doi:10.1161/JAHA.119.012177 31500474 PMC6818002

[7] Joosten MM, Pai JK, Bertoia ML, . Associations between conventional cardiovascular risk factors and risk of peripheral artery disease in men. JAMA. 2012;308(16):1660-1667. doi:10.1001/jama.2012.13415 23093164 PMC3733106

[8] Kannel WB, McGee DL. Update on some epidemiologic features of intermittent claudication: the Framingham Study. J Am Geriatr Soc. 1985;33(1):13-18. doi:10.1111/j.1532-5415.1985.tb02853.x 3965550

[9] Meijer WT, Grobbee DE, Hunink MG, Hofman A, Hoes AW. Determinants of peripheral arterial disease in the elderly: the Rotterdam Study. Arch Intern Med. 2000;160(19):2934-2938. doi:10.1001/archinte.160.19.2934 11041900

[10] Murabito JM, Evans JC, Nieto K, Larson MG, Levy D, Wilson PW. Prevalence and clinical correlates of peripheral arterial disease in the Framingham Offspring Study. Am Heart J. 2002;143(6):961-965. doi:10.1067/mhj.2002.122871 12075249

[11] Gerhard-Herman MD, Gornik HL, Barrett C, . 2016 AHA/ACC guideline on the management of patients with lower extremity peripheral artery disease: executive summary: a report of the American College of Cardiology/American Heart Association Task Force on Clinical Practice Guidelines. Circulation. 2017;135(12):e686-e725. doi:10.1161/CIR.0000000000000470 27840332 PMC5479414

[12] Stoffers HE, Rinkens PE, Kester AD, Kaiser V, Knottnerus JA. The prevalence of asymptomatic and unrecognized peripheral arterial occlusive disease. Int J Epidemiol. 1996;25(2):282-290. doi:10.1093/ije/25.2.282 9119553

[13] Newman AB, Siscovick DS, Manolio TA, ; Cardiovascular Heart Study (CHS) Collaborative Research Group. Ankle-arm index as a marker of atherosclerosis in the Cardiovascular Health Study. Circulation. 1993;88(3):837-845. doi:10.1161/01.CIR.88.3.837 8353913

[14] Allison MA, Criqui MH, McClelland RL, . The effect of novel cardiovascular risk factors on the ethnic-specific odds for peripheral arterial disease in the Multi-Ethnic Study of Atherosclerosis (MESA). J Am Coll Cardiol. 2006;48(6):1190-1197. doi:10.1016/j.jacc.2006.05.049 16979004

[15] Zheng ZJ, Sharrett AR, Chambless LE, . Associations of ankle-brachial index with clinical coronary heart disease, stroke and preclinical carotid and popliteal atherosclerosis: the Atherosclerosis Risk in Communities (ARIC) Study. Atherosclerosis. 1997;131(1):115-125. doi:10.1016/S0021-9150(97)06089-9 9180252

[16] Collins TC, Petersen NJ, Suarez-Almazor M, Ashton CM. The prevalence of peripheral arterial disease in a racially diverse population. Arch Intern Med. 2003;163(12):1469-1474. doi:10.1001/archinte.163.12.1469 12824097

[17] Mohler ER III, Bundens W, Denenberg J, Medenilla E, Hiatt WR, Criqui MH. Progression of asymptomatic peripheral artery disease over 1 year. Vasc Med. 2012;17(1):10-16. doi:10.1177/1358863X11431106 22363014

[18] Kennedy M, Solomon C, Manolio TA, . Risk factors for declining ankle-brachial index in men and women 65 years or older: the Cardiovascular Health Study. Arch Intern Med. 2005;165(16):1896-1902. doi:10.1001/archinte.165.16.1896 16157835

[19] Sarkar S, Esserman DA, Skanderson M, Levin FL, Justice AC, Lim JK. Disparities in hepatitis C testing in U.S. veterans born 1945-1965. J Hepatol. 2016;65(2):259-265. doi:10.1016/j.jhep.2016.04.012 27130843 PMC4955712

[20] Njei B, Esserman D, Krishnan S, . Regional and rural-urban differences in the use of direct-acting antiviral agents for hepatitis C virus: the Veteran Birth Cohort. Med Care. 2019;57(4):279-285. doi:10.1097/MLR.0000000000001071 30807449 PMC6436819

[21] Bali V, Yermilov I, Coutts K, Legorreta AP. Novel screening metric for the identification of at-risk peripheral artery disease patients using administrative claims data. Vasc Med. 2016;21(1):33-40. doi:10.1177/1358863X15616687 26608733

[22] Hernandez SE, Sylling PW, Mor MK, . Developing an algorithm for combining race and ethnicity data sources in the Veterans Health Administration. Mil Med. 2020;185(3-4):e495-e500. doi:10.1093/milmed/usz322 31603222

[23] Hamilton NS, Edelman D, Weinberger M, Jackson GL. Concordance between self-reported race/ethnicity and that recorded in a Veteran Affairs electronic medical record. N C Med J. 2009;70(4):296-300. doi:10.18043/ncm.70.4.296 19835243

[24] Beckman JA, Duncan MS, Alcorn CW, . Association of human immunodeficiency virus infection and risk of peripheral artery disease. Circulation. 2018;138(3):255-265. doi:10.1161/CIRCULATIONAHA.117.032647 29535090 PMC6050082

[25] McGinnis KA, Brandt CA, Skanderson M, . Validating smoking data from the Veteran’s Affairs Health Factors dataset, an electronic data source. Nicotine Tob Res. 2011;13(12):1233-1239. doi:10.1093/ntr/ntr206 21911825 PMC3223583

[26] Klarin D, Lynch J, Aragam K, ; VA Million Veteran Program. Genome-wide association study of peripheral artery disease in the Million Veteran Program. Nat Med. 2019;25(8):1274-1279. doi:10.1038/s41591-019-0492-5 31285632 PMC6768096

[27] Meadows M, Peterson A, Boyko EJ, Littman AJ. Validity of methods to identify individuals with lower extremity amputation using Department of Veterans Affairs electronic medical records. Arch Rehabil Res Clin Transl. 2022;4(1):100182. doi:10.1016/j.arrct.2022.100182 35282148 PMC8904866

[28] Freiberg MS, Chang C-CH, Kuller LH, . HIV infection and the risk of acute myocardial infarction. JAMA Intern Med. 2013;173(8):614-622. doi:10.1001/jamainternmed.2013.3728 23459863 PMC4766798

[29] Little RJA, Rubin DB. Statistical Analysis With Missing Data. 3rd ed. John Wiley and Sons; 2019.

[30] Schafer JL. Analysis of Incomplete Multivariate Data. Chapman and Hall; 1997. doi:10.1201/9781439821862

[31] Rubin DB. Multiple Imputation for Nonresponse in Surveys. John Wiley and Sons; 1987. doi:10.1002/9780470316696

[32] Austin PC, Lee DS, Fine JP. Introduction to the analysis of survival data in the presence of competing risks. Circulation. 2016;133(6):601-609. doi:10.1161/CIRCULATIONAHA.115.017719 26858290 PMC4741409

[33] Presser L. The Black American amputation epidemic. ProPublica. Accessed January 15, 2021. https://features.propublica.org/diabetes-amputations/black-american-amputation-epidemic/

[34] Criqui MH, Vargas V, Denenberg JO, . Ethnicity and peripheral arterial disease: the San Diego Population Study. Circulation. 2005;112(17):2703-2707. doi:10.1161/CIRCULATIONAHA.105.546507 16246968

[35] Arya S, Khakharia A, Binney ZO, . Association of statin dose with amputation and survival in patients with peripheral artery disease. Circulation. 2018;137(14):1435-1446. doi:10.1161/CIRCULATIONAHA.117.032361 29330214 PMC5882502

[36] Willey J, Mentias A, Vaughan-Sarrazin M, McCoy K, Rosenthal G, Girotra S. Epidemiology of lower extremity peripheral artery disease in veterans. J Vasc Surg. 2018;68(2):527-535.e5. doi:10.1016/j.jvs.2017.11.083 29588132 PMC6132057

[37] Arya S, Binney Z, Khakharia A, . Race and socioeconomic status independently affect risk of major amputation in peripheral artery disease. J Am Heart Assoc. 2018;7(2):e007425. doi:10.1161/JAHA.117.007425 29330260 PMC5850162

[38] Fowkes FG, Housley E, Riemersma RA, . Smoking, lipids, glucose intolerance, and blood pressure as risk factors for peripheral atherosclerosis compared with ischemic heart disease in the Edinburgh Artery Study. Am J Epidemiol. 1992;135(4):331-340. doi:10.1093/oxfordjournals.aje.a116294 1550087

[39] Gates MA, Holowka DW, Vasterling JJ, Keane TM, Marx BP, Rosen RC. Posttraumatic stress disorder in veterans and military personnel: epidemiology, screening, and case recognition. Psychol Serv. 2012;9(4):361-382. doi:10.1037/a0027649 23148803

