# Supplementary material for: Association of Sex and Race With Incident Peripheral Artery Disease Among Veterans With Normal Ankle-Brachial Indices
Source: JAMA Netw Open. 2022 Nov 3;5(11):e2240188. doi: 10.1001/jamanetworkopen.2022.40188 (PMC9634499; doi:10.1001/jamanetworkopen.2022.40188)
Supplement: Supplement. — eTable 1. Diagnostic and Procedural Codes for Peripheral Artery Disease eTable 2. Baseline Characteristics of Individuals Excluded From the Study eTable 3. Procedural Codes for Lower-Extremity Amputation Due to Trauma or Non–Peripheral Artery Disease Indications eTable 4. Association of Sex, Race, and Ethnicity With Peripheral Artery Disease Among Individuals Without Diabetes eTable 5. Association of Sex, Race, and Ethnicity With Peripheral Artery Disease Among Individual Who Never Smoked eTable 6. Association of Sex, Race, and Ethnicity With Peripheral Artery Disease Among Individuals Without Diabetes Who Never Smoked eTable 7. Baseline Characteristics of Individuals Without Diabetes Who Never Smoked eTable 8. Deaths During Follow-up by Sex, Race and Ethnicity, Diabetes, and Smoking eTable 9. Association of Sex, Race and Ethnicity, Diabetes, and Smoking With Subsequent Ankle-Brachial Index Less Than 0.90 eFigure 1. Sample Derivation eFigure 2. Cumulative Incidence of Peripheral Artery Disease by History of Diabetes eFigure 3. Cumulative Incidence of Peripheral Artery Disease by Smoking History eFigure 4. Distribution of Time to Incident Peripheral Artery Disease by Race [file jamanetwopen-e2240188-s001.pdf]

Supplemental Online Content

Aday AW, Duncan MS, Patterson OV, et al. Association of sex and race with incident peripheral artery disease among veterans with normal ankle-brachial indices. *JAMA Netw Open.* 2022;5(11):e2240188. doi:10.1001/jamanetworkopen.2022.40188

eTable 1. Diagnostic and Procedural Codes for Peripheral Artery Disease
eTable 2. Baseline Characteristics of Individuals Excluded From the Study
eTable 3. Procedural Codes for Lower-Extremity Amputation Due to Trauma or Non–Peripheral Artery Disease Indications
eTable 4. Association of Sex, Race, and Ethnicity With Peripheral Artery Disease Among Individuals Without Diabetes
eTable 5. Association of Sex, Race, and Ethnicity With Peripheral Artery Disease Among Individual Who Never Smoked
eTable 6. Association of Sex, Race, and Ethnicity With Peripheral Artery Disease Among Individuals Without Diabetes Who Never Smoked
eTable 7. Baseline Characteristics of Individuals Without Diabetes Who Never Smoked
eTable 8. Deaths During Follow-up by Sex, Race and Ethnicity, Diabetes, and Smoking
eTable 9. Association of Sex, Race and Ethnicity, Diabetes, and Smoking With Subsequent Ankle-Brachial Index Less Than 0.90
eFigure 1. Sample Derivation
eFigure 2. Cumulative Incidence of Peripheral Artery Disease by History of Diabetes
eFigure 3. Cumulative Incidence of Peripheral Artery Disease by Smoking History
eFigure 4. Distribution of Time to Incident Peripheral Artery Disease by Race

This supplemental material has been provided by the authors to give readers additional information about their work.

© 2022 Aday AW et al. *JAMA Network Open.*

eTable 1. Diagnostic and Procedural Codes fFor Peripheral Artery Disease

Description	ICD-9 Procedure Codes	CPT Codes	ICD-10 Procedure Codes
Lower Extremity Amputation	84.10, 84.11, 84.12, 84.13, 84.14, 84.15, 84.16, 84.17, 84.18, 84.19, 84.91	27295, 27590, 27591, 27592, 27598, 27880, 27881, 27882, 27888, 27889, 28800, 28805	0Y6C0Z1, 0Y6C0Z2, 0Y6C0Z3, 0Y6D0Z1, 0Y6D0Z2, 0Y6D0Z3, 0Y6H0Z1, 0Y6H0Z2, 0Y6H0Z3, 0Y6J0Z1, 0Y6J0Z2, 0Y6J0Z3, 0Y6P0Z0, 0Y6P0Z10, Y6P0Z2, 0Y6P0Z3, 0Y6Q0Z0, 0Y6Q0Z1, 0Y6Q0Z2, 0Y6Q0Z3, 0Y6R0Z0, 0Y6R0Z1, 0Y6R0Z2, 0Y6R0Z3, 0Y6S0Z0, 0Y6S0Z1, 0Y6S0Z2, 0Y6S0Z3, 0Y6T0Z0, 0Y6T0Z1, 0Y6T0Z2, 0Y6T0Z3, 0Y6U0Z0, 0Y6U0Z1, 0Y6U0Z2, 0Y6U0Z3, 0Y6V0Z0, 0Y6V0Z1, 0Y6V0Z2, 0Y6V0Z3, 0Y6W0Z0, 0Y6W0Z1, 0Y6W0Z2, 0Y6W0Z3, 0Y6X0Z0, 0Y6X0Z1, 0Y6X0Z2, 0Y6X0Z3, 0Y6Y0Z0, 0Y6Y0Z1, 0Y6Y0Z2, 0Y6Y0Z3, 0Y6M0Z4, 0Y6M0Z5, 0Y6M0Z6, 0Y6M0Z7, 0Y6M0Z8, 0Y6M0Z9, 0Y6M0ZB, 0Y6M0ZC, 0Y6M0ZD, 0Y6M0ZF, 0Y6N0Z4, 0Y6N0Z5, 0Y6N0Z6, 0Y6N0Z7, 0Y6N0Z8, 0Y6N0Z9, 0Y6N0ZB, 0Y6N0ZC, 0Y6N0ZD, 0Y6N0ZF, 0Y6M0Z0, 0Y6N0Z0, 0Y6H0Z3, 0Y6J0Z3, 0Y6H0Z1, 0Y6H0Z2, 0Y6H0Z3, 0Y6J0Z1, 0Y6J0Z2, 0Y6J0Z3, 0Y6F0ZZ, 0Y6G0ZZ, 0Y6C0Z1, 0Y6C0Z2, 0Y6C0Z3, 0Y6D0Z1, 0Y6D0Z2, 0Y6D0Z3, 0Y670ZZ, 0Y680ZZ, 0Y620ZZ, 0Y630ZZ, 0Y640ZZ, 0Y620ZZ, 0Y630ZZ, 0Y640ZZ
Lower Extremity Peripheral Angioplasty, Atherectomy, or Endarterectomy	38.88, 39.50	35302, 35303, 35304, 35305, 35306, 35331, 35351, 35355, 35361, 35363, 35371, 35372, 35381, 35452, 35454, 35456, 35459, 35470, 35472, 35473, 35474, 35481, 35482, 35483, 35485, 35491, 35492, 35493, 35495	04LK0CZ, 04LK0DZ, 04LK0ZZ, 04LK3CZ, 04LK3DZ, 04LK3ZZ, 04LK4CZ, 04LK4DZ, 04LK4ZZ, 04LL0CZ, 04LL0DZ, 04LL0ZZ, 04LL3CZ, 04LL3DZ, 04LL3ZZ, 04LL4CZ, 04LL4DZ, 04LL4ZZ, 04LM0CZ, 04LM0DZ, 04LM0ZZ, 04LM3CZ, 04LM3DZ, 04LM3ZZ, 04LM4CZ, 04LM4DZ, 04LM4ZZ, 04LN0CZ, 04LN0DZ, 04LN0ZZ, 04LN3CZ, 04LN3DZ, 04LN3ZZ, 04LN4CZ, 04LN4DZ, 04LN4ZZ, 04LP0CZ, 04LP0DZ, 04LP0ZZ, 04LP3CZ, 04LP3DZ, 04LP3ZZ, 04LP4CZ, 04LP4DZ, 04LP4ZZ, 04LQ0CZ, 04LQ0DZ, 04LQ0ZZ, 04LQ3CZ, 04LQ3DZ, 04LQ3ZZ, 04LQ4CZ, 04LQ4DZ, 04LQ4ZZ, 04LR0CZ, 04LR0DZ, 04LR0ZZ, 04LR3CZ, 04LR3DZ, 04LR3ZZ, 04LR4CZ, 04LR4DZ, 04LR4ZZ, 04LS0CZ, 04LS0DZ, 04LS0ZZ, 04LS3CZ, 04LS3DZ, 04LS3ZZ, 04LS4CZ, 04LS4DZ, 04LS4ZZ, 04LT0CZ, 04LT0DZ, 04LT0ZZ, 04LT3CZ, 04LT3DZ, 04LT3ZZ, 04LT4CZ, 04LT4DZ, 04LT4ZZ, 04LU0CZ, 04LU0DZ, 04LU0ZZ, 04LU3CZ, 04LU3DZ, 04LU3ZZ, 04LU4CZ, 04LU4DZ, 04LU4ZZ, 04LV0CZ, 04LV0DZ, 04LV0ZZ, 04LV3CZ, 04LV3DZ, 04LV3ZZ, 04LV4CZ, 04LV4DZ, 04LV4ZZ, 04LW0CZ, 04LW0DZ, 04LW0ZZ, 04LW3CZ, 04LW3DZ, 04LW3ZZ, 04LW4CZ, 04LW4DZ, 04LW4ZZ, 047C041, 047C04Z, 047C0D1, 047C0DZ, 047C0Z1, 047C0ZZ, 047C341, 047C34Z, 047C3D1, 047C3DZ, 047C3Z1, 047C3ZZ, 047C441, 047C44Z, 047C4D1, 047C4DZ, 047C4Z1, 047C4ZZ, 047D041, 047D04Z, 047D0D1, 047D0DZ, 047D0Z1, 047D0ZZ, 047D341, 047D34Z, 047D3D1, 047D3DZ, 047D3Z1, 047D3ZZ, 047D441, 047D44Z, 047D4D1, 047D4DZ, 047D4Z1, 047D4ZZ, 047E041, 047E04Z, 047E0D1, 047E0DZ, 047E0Z1, 047E0ZZ, 047E341, 047E34Z, 047E3D1, 047E3DZ, 047E3Z1, 047E3ZZ, 047E441, 047E44Z, 047E4D1, 047E4DZ, 047E4Z1, 047E4ZZ, 047F041, 047F04Z, 047F0D1, 047F0DZ, 047F0Z1, 047F0ZZ, 047F341, 047F34Z, 047F3D1, 047F3DZ, 047F3Z1, 047F3ZZ, 047F441, 047F44Z, 047F4D1, 047F4DZ, 047F4Z1, 047F4ZZ, 047H041, 047H04Z, 047H0D1, 047H0DZ,

© 2022 Aday AW et al. *JAMA Network Open.*

Description	ICD-9 Procedure Codes	CPT Codes	ICD-10 Procedure Codes
			047H0Z1, 047H0ZZ, 047H341, 047H34Z, 047H3D1, 047H3DZ, 047H3Z1, 047H3ZZ, 047H441, 047H44Z, 047H4D1, 047H4DZ, 047H4Z1, 047H4ZZ, 047J041, 047J04Z, 047J0D1, 047J0DZ, 047J0Z1, 047J0ZZ, 047J341, 047J34Z, 047J3D1, 047J3DZ, 047J3Z1, 047J3ZZ, 047J441, 047J44Z, 047J4D1, 047J4DZ, 047J4Z1, 047J4ZZ, 047K04Z, 047K0DZ, 047K0ZZ, 047K34Z, 047K3DZ, 047K3ZZ, 047K44Z, 047K4DZ, 047K4ZZ, 047L04Z,, 047L0ZZ, 047L34Z, 047L3DZ, 047L3ZZ, 047L44Z, 047L4DZ, 047L4ZZ, 047M04Z, 047M0DZ, 047M0ZZ, 047M34Z, 047M3DZ, 047M3ZZ, 047M44Z, 047M4DZ, 047M4ZZ, 047N04Z, 047N0DZ, 047N0ZZ, 047N34Z, 047N3DZ, 047N3ZZ, 047N44Z, 047N4DZ, 047N4ZZ, 047N04Z, 047N0DZ, 047N0ZZ, 047N34Z, 047N3DZ, 047N3ZZ, 047N44Z, 047N4DZ, 047N4ZZ, 047P041, 047P04Z, 047P0D1, 047P0DZ, 047P0Z1, 047P0ZZ, 047P341, 047P34Z, 047P3D1, 047P3DZ, 047P3Z1, 047P3ZZ, 047P441, 047P44Z, 047P4D1, 047P4DZ, 047P4Z1, 047P4ZZ, 047Q041, 047Q04Z, 047Q0D1, 047Q0DZ, 047Q0Z1, 047Q0ZZ, 047Q341, 047Q34Z, 047Q3D1, 047Q3DZ, 047Q3Z1, 047Q3ZZ, 047Q441, 047Q44Z, 047Q4D1, 047Q4DZ, 047Q4Z1, 047Q4ZZ, 047R041, 047R04Z, 047R0D1, 047R0DZ, 047R0Z1, 047R0ZZ, 047R34Z, 047R3D1, 047R3DZ, 047R3Z1, 047R3ZZ, 047R441, 047R44Z, 047R4D1, 047R4DZ, 047R4Z1, 047R4ZZ, 047S041, 047S04Z, 047S0D1, 047S0DZ, 047S0Z1, 047S0ZZ, 047S341, 047S34Z, 047S3D1, 047S3DZ, 047S3Z1, 047S3ZZ,, 047S441, 047S44Z, 047S4D1, 047S4DZ, 047S4Z1, 047S4ZZ, 047T041, 047T04Z, 047T0D1, 047T0DZ, 047T0Z1, 047T0ZZ, 047T341, 047T34Z, 047T3D1, 047T3DZ, 047T3Z1, 047T3ZZ, 047T441, 047T44Z,, 047T4DZ, 047T4Z1, 047T4ZZ, 047U041, 047U04Z, 047U0D1,, 047U0Z1, 047U0ZZ, 047U341, 047U34Z, 047U3D1, 047U3DZ, 047U3Z1, 047U3ZZ, 047U441, 047U44Z, 047U4D1, 047U4DZ, 047U4Z1, 047U4ZZ, 047V041, 047V04Z, 047V0D1, 047V0DZ, 047V0Z1, 047V0ZZ, 047V341, 047V34Z, 047V3D1, 047V3DZ, 047V3Z1, 047V3ZZ, 047V441, 047V44Z, 047V4D1, 047V4DZ, 047V4Z1, 047V4ZZ, 047W041, 047W04Z, 047W0D1, 047W0DZ, 047W0Z1, 047W0ZZ, 047W341, 047W34Z, 047W3D1, 047W3DZ, 047W3Z1, 047W3ZZ, 047W441, 047W44Z, 047W4D1, 047W4DZ, 047W4Z1, 047W4ZZ, 047Y041, 047Y04Z, 047Y0D1, 047Y0DZ, 047Y0Z1, 047Y0ZZ, 047Y341, 047Y34Z, 047Y3D1, 047Y3DZ, 047Y3Z1, 047Y3ZZ, 047Y441, 047Y44Z, 047Y4D1, 047Y4DZ, 047Y4Z1, 047Y4ZZ
Lower Extremity Peripheral Bypass	39.25, 39.29	35521, 35533, 35537, 35538, 35539, 35540, 35541, 35546, 35548, 35549, 35551, 35556, 35558, 35563,	0410096, 0410097, 0410098, 0410099, 041009B, 041009C, 041009D, 041009F, 041009G, 041009H, 041009J, 041009K, 041009Q, 041009R, 04100A6, 04100A7, 04100A8, 04100A9, 04100AB, 04100AC, 04100AD, 04100AF, 04100AG, 04100AH, 04100AJ, 04100AK, 04100AQ, 04100AR, 04100J6, 04100J7, 04100J8, 04100J9, 04100JB, 04100JC, 04100JD, 04100JF, 04100JG, 04100JH, 04100JJ, 04100JK, 04100JQ, 04100JR, 04100K6, 04100K7, 04100K8, 04100K9, 04100KB, 04100KC, 04100KD, 04100KF, 04100KG, 04100KH, 04100KJ, 04100KK, 04100KQ, 04100KR, 04100Z6, 04100Z7, 04100Z8,

© 2022 Aday AW et al. *JAMA Network Open.*

Description	ICD-9 Procedure Codes	CPT Codes	ICD-10 Procedure Codes
		35565, 35566, 35571, 35582, 35583, 35585, 35587, 35621, 35623, 35637, 35638, 35641, 35646, 35647, 35651, 35654, 35656, 35661, 35663, 35665, 35666, 35671	04100Z9, 04100ZB, 04100ZC, 04100ZD, 04100ZF, 04100ZG, 04100ZH, 04100ZJ, 04100ZK, 04100ZQ, 04100ZR, 0410496, 0410497, 0410498, 0410499, 041049B, 041049C, 041049D, 041049F, 041049G, 041049H, 041049J, 041049K, 041049Q, 041049R, 04104A6, 04104A7, 04104A8, 04104AA, 04104AB, 04104AC, 04104AD, 04104AF, 04104AG, 04104AH, 04104AJ, 04104AK, 04104AQ, 04104AR, 04100ZR, 04104J6, 04104J7, 04104J8, 04104JJ, 04104JB, 04104JC, 04104JD, 04104JF, 04104JG, 04104JH, 04104JJ, 04104JK, 04104JQ, 04104JR, 04100ZR, 04104K6, 04104K7, 04104K8, 04104KK, 04104KB, 04104KC, 04104KD, 04104KF, 04104KG, 04104KH, 04104KJ, 04104KK, 04104KQ, 04104KR, 04100ZR, 04104Z6, 04104Z7, 04104Z8, 04104ZZ, 04104ZB, 04104ZC, 04104ZD, 04104ZF, 04104ZG, 04104ZH, 04104ZJ, 04104ZK, 04104ZQ, 04104ZR, 041C09H, 041C09J, 041C09K, 041C0AH, 041C0AJ, 041C0AK, 041C0JH, 041C0JJ, 041C0JK, 041C0KH, 041C0KJ, 041C0KK, 041C0ZH, 041C0ZJ, 041C0ZK, 041C49H, 041C49J, 041C49K, 041C4AH, 041C4AJ, 041C4AK, 041C4JH, 041C4JJ, 041C4JK, 041C4KH, 041C4KJ, 041C4KK, 041C4ZH, 041C4ZJ, 041C4ZK, 041D09H, 041D09J, 041D09K, 041D0AH, 041D0AJ, 041D0AK, 041D0JH, 041D0JJ, 041D0JK, 041D0KH, 041D0KJ, 041D0KK, 041D0ZH, 041D0ZJ, 041D0ZK, 041D49H, 041D49J, 041D49K, 041D4AH, 041D4AJ, 041D4AK, 041D4JH, 041D4JJ, 041D4JK, 041D4KH, 041D4KJ, 041D4KK, 041D4ZH, 041D4ZJ, 041D4ZK, 041E09H, 041E09J, 041E09K, 041E0AH, 041E0AJ, 041E0AK, 041E0JH, 041E0JJ, 041E0JK, 041E0KH, 041E0KJ, 041E0KK, 041E0ZH, 041E0ZJ, 041E0ZK, 041E49H, 041E49J, 041E49K, 041E4AH, 041E4AJ, 041E4AK, 041E4JH, 041E4JJ, 041E4JK, 041E4KH, 041E4KJ, 041E4KK, 041E4ZH, 041E4ZJ, 041E4ZK, 041F09H, 041F09J, 041F09K, 041F0AH, 041F0AJ, 041F0AK, 041F0JH, 041F0JJ, 041F0JK, 041F0KH, 041F0KJ, 041F0KK, 041F0ZH, 041F0ZJ, 041F0ZK, 041F49H, 041F49J, 041F49K, 041F4AH, 041F4AJ, 041F4AK, 041F4JH, 041F4JJ, 041F4JK, 041F4KH, 041F4KJ, 041F4KK, 041F4ZH, 041F4ZJ, 041F4ZK, 041H09H, 041H09J, 041H09K, 041H0AH, 041H0AJ, 041H0AK, 041H0JH, 041H0JJ, 041H0JK, 041H0KH, 041H0KJ, 041H0KK, 041H0ZH, 041H0ZJ, 041H0ZK, 041H49H, 041H49J, 041H49K, 041H4AH, 041H4AJ, 041H4AK, 041H4JH, 041H4JJ, 041H4JK, 041H4KH, 041H4KJ, 041H4KK, 041H4ZH, 041H4ZJ, 041H4ZK, 041J09H, 041J09J, 041J09K, 041J0AH, 041J0AJ, 041J0AK, 041J0JH, 041J0JJ, 041J0JK, 041J0KH, 041J0KJ, 041J0KK, 041J0ZH, 041J0ZJ, 041J0ZK, 041J49H, 041J49J, 041J49K, 041J4AH, 041J4AJ, 041J4AK, 041J4JH, 041J4JJ, 041J4JK, 041J4KH, 041J4KJ, 041J4KK, 041J4ZH, 041J4ZJ, 041J4ZK, 0312095, 0312096, 0312097, 0312098, 0312099, 031209B, 031209C, 03120A6, 03120A7, 03120A8, 03120A9, 03120AB, 03120AC, 03120J6, 03120J7, 03120J8, 03120J9, 03120JB, 03120JC, 03120K6, 03120K7, 03120K8, 03120K9, 03120KB, 03120KC, 03120Z6, 03120Z7, 03120Z8, 03120Z9, 03120ZB, 03120ZC, 0313096, 0313097, 0313098, 0313099, 031309B, 031309C, 03130A6, 03130A7, 03130A8,

Description	ICD-9 Procedure Codes	CPT Codes	ICD-10 Procedure Codes
			03130A9, 03130AB, 03130AC, 03130J6, 03130J7, 03130J8, 03130J9, 03130JB, 03130JC, 03130K6, 03130K7, 03130K8, 03130K9, 03130KB, 03130KC, 03130Z6, 03130Z7, 03130Z8, 03130Z9, 03130ZB, 03130ZC, 0314096, 0314097, 0314098, 0314099, 031409B, 031409C, 03140A6, 03140A7, 03140A8, 03140A9, 03140AB, 03140AC, 03140J6, 03140J7, 03140J8, 03140J9, 03140JB, 03140JC, 03140K6, 03140K7, 03140K8, 03140K9, 03140KB, 03140KC, 03140Z6, 03140Z7, 03140Z8, 03140Z9, 03140ZB, 03140ZC, 0315096, 0315097, 0315098, 0315099, 031509B, 031509C, 03150A6, 03150A7, 03150A8, 03150A9, 03150AB, 03150AC, 03150J6, 03150J7, 03150J8, 03150J9, 03150JB, 03150JC, 03150K6, 03150K7, 03150K8, 03150K9, 03150KB, 03150KC, 03150Z6, 03150Z7, 03150Z8, 03150Z9, 03150ZB, 03150ZC, 0316096, 0316097, 0316098, 0316099, 031609B, 031609C, 03160A6, 03160A7, 03160A8, 03160A9, 03160AB, 03160AC, 03160J6, 03160J7, 03160J8, 03160J9, 03160JB, 03160JC, 03160K6, 03160K7, 03160K8, 03160K9, 03160KB, 03160KC, 03160Z6, 03160Z7, 03160Z8, 03160Z9, 03160ZB, 03160ZC, 041K09H, 041K09J, 041K09K, 041K09L, 041K09M, 041K09N, 041K09P, 041K09Q, 041K09S, 041K0AH, 041K0AJ, 041K0AK, 041K0AL, 041K0AM, 041K0AN, 041K0AP, 041K0AQ, 041K0AS, 041K0JH, 041K0JJ, 041K0JK, 041K0JL, 041K0JM, 041K0JN, 041K0JP, 041K0JQ, 041K0JS, 041K0KH, 041K0KJ, 041K0KK, 041K0KL, 041K0KM, 041K0KN, 041K0KP, 041K0KQ, 041K0KS, 041K0ZH, 041K0ZJ, 041K0ZK, 041K0ZL, 041K0ZM, 041K0ZN, 041K0ZP, 041K0ZQ, 041K0ZS, 041K49H, 041K49J, 041K49K, 041K49L, 041K49M, 041K49N, 041K49P, 041K49Q, 041K49S, 041K4AH, 041K4AJ, 041K4AK, 041K4AL, 041K4AM, 041K4AN, 041K4AP, 041K4AQ, 041K4AS, 041K4JH, 041K4JJ, 041K4JK, 041K4JL, 041K4JM, 041K4JN, 041K4JP, 041K4JQ, 041K4JS, 041K4KH, 041K4KJ, 041K4KK, 041K4KL, 041K4KM, 041K4KN, 041K4KP, 041K4KQ, 041K4KS, 041K4ZH, 041K4ZJ, 041K4ZK, 041K4ZL, 041K4ZM, 041K4ZN, 041K4ZP, 041K4ZQ, 041K4ZS, 041L09H, 041L09J, 041L09K, 041L09L, 041L09M, 041L09N, 041L09P, 041L09Q, 041L09S, 041L0AH, 041L0AJ, 041L0AK, 041L0AL, 041L0AM, 041L0AN, 041L0AP, 041L0AQ, 041L0AS, 041L0JH, 041L0JJ, 041L0JK, 041L0JL, 041L0JM, 041L0JN, 041L0JP, 041L0JQ, 041L0JS, 041L0KH, 041L0KJ, 041L0KK, 041L0KL, 041L0KM, 041L0KN, 041L0KP, 041L0KQ, 041L0KS, 041L0ZH, 041L0ZJ, 041L0ZK, 041L0ZL, 041L0ZM, 041L0ZN, 041L0ZP, 041L0ZQ, 041L0ZS, 041L49H, 041L49J, 041L49K, 041L49L, 041L49M, 041L49N, 041L49P, 041L49Q, 041L49S, 041L4AH, 041L4AJ, 041L4AK, 041L4AL, 041L4AM, 041L4AN, 041L4AP, 041L4AQ, 041L4AS, 041L4JH, 041L4JJ, 041L4JK, 041L4JL, 041L4JM, 041L4JN, 041L4JP, 041L4JQ, 041L4JS, 041L4KH, 041L4KJ, 041L4KK, 041L4KL, 041L4KM, 041L4KN, 041L4KP, 041L4KQ, 041L4KS, 041L4ZH, 041L4ZJ, 041L4ZK, 041L4ZL, 041L4ZM, 041L4ZN, 041L4ZP, 041L4ZQ, 041L4ZS, 041M09L, 041M09M, 041M09P, 041M09Q, 041M09S, 041M0AL, 041M0AM, 041M0AP, 041M0AQ, 041M0AS, 041M0JL, 041M0JM, 041M0JP,

Description	ICD-9 Procedure Codes	CPT Codes	ICD-10 Procedure Codes
			041M0JQ, 041M0JS, 041M0KL, 041M0KM, 041M0KP, 041M0KQ, 041M0KS, 041M0ZL, 041M0ZM, 041M0ZP, 041M0ZQ, 041M0ZS, 041M49L, 041M49M, 041M49P, 041M49Q, 041M49S, 041M4AL, 041M4AM, 041M4AP, 041M4AQ, 041M4AS, 041M4JL, 041M4JM, 041M4JP, 041M4JQ, 041M4JS, 041M4KL, 041M4KM, 041M4KP, 041M4KQ, 041M4KS, 041M4ZL, 041M4ZM, 041M4ZP, 041M4ZQ, 041M4ZS, 041N09L, 041N09M, 041N09P, 041N09Q, 041N09S, 041N0AL, 041N0AM, 041N0AP, 041N0AQ, 041N0AS, 041N0JL, 041N0JM, 041N0JP, 041N0JQ, 041N0JS, 041N0KL, 041N0KM, 041N0KP, 041N0KQ, 041N0KS, 041N0ZL, 041N0ZM, 041N0ZP, 041N0ZQ, 041N0ZS, 041N49L, 041N49M, 041N49P, 041N49Q, 041N49S, 041N4AL, 041N4AM, 041N4AP, 041N4AQ, 041N4AS, 041N4JL, 041N4JM, 041N4JP, 041N4JQ, 041N4JS, 041N4KL, 041N4KM, 041N4KP, 041N4KQ, 041N4KS, 041N4ZL, 041N4ZM, 041N4ZP, 041N4ZQ, 041N4ZS, 041T09P, 041T09Q, 041T09S, 041T0AP, 041T0AQ, 041T0AS, 041T0JP, 041T0JQ, 041T0JS, 041T0KP, 041T0KQ, 041T0KS, 041T0ZP, 041T0ZQ, 041T0ZS, 041T49P, 041T49Q, 041T49S, 041T4AP, 041T4AQ, 041T4AS, 041T4JP, 041T4JQ, 041T4JS, 041T4KP, 041T4KQ, 041T4KS, 041T4ZP, 041T4ZQ, 041T4ZS, 041U09P, 041U09Q, 041U09S, 041U0AP, 041U0AQ, 041U0AS, 041U0JP, 041U0JQ, 041U0JS, 041U0KP, 041U0KQ, 041U0KS, 041U0ZP, 041U0ZQ, 041U0ZS, 041U49P, 041U49Q, 041U49S, 041U4AP, 041U4AQ, 041U4AS, 041U4JP, 041U0JQ, 041U4JS, 041U0KP, 041U4KQ, 041U4KS, 041U4ZP, 041U4ZQ, 041U4ZS, 041V09P, 041V09Q, 041V09S, 041V0AP, 041V0AQ, 041V0AS, 041V0JP, 041V0JQ, 041V0JS, 041V0KP, 041V0KQ, 041V0KS, 041V0ZP, 041V0ZQ, 041V0ZS, 041V49P, 041V49Q, 041V49S, 041V4AP, 041V4AQ, 041V4AS, 041V4JP, 041V4JQ, 041V4JS, 041V4KP, 041V4KQ, 041V4KS,, 041V4ZQ, 041V4ZS, 041W09P, 041W09Q, 041W09S, 041W0AP, 041W0AQ, 041W0AS, 041W0JP, 041W0JQ, 041W0JS, 041W0KP, 041W0KQ, 041W0KS, 041W0ZP, 041W0ZQ,, 041W49P, 041W49Q, 041W49S, 041W4AP, 041W4AQ, 041W4AS, 041W4JP, 041W4JQ, 041W4JS, 041W4KP, 041W4KQ, 041W4KS, 041W4ZP, 041W4ZQ, 041W4ZS
Repair, Exploration, Revision, Resection of Lower Extremity Arteries, or Thrombectomy of Graft	38.08, 38.38, 38.48, 39.49, 39.56, 39.57, 39.58	35226, 35256, 35286, 35700, 35721, 35741, 35876, 35879, 35881, 35883, 35884	04CK0ZZ, 04CK4ZZ, 04CL0ZZ, 04CL4ZZ, 04CM0ZZ, 04CM4ZZ, 04CN0ZZ, 04CN4ZZ, 04CP0ZZ, 04CP4ZZ, 04CQ0ZZ, 04CQ4ZZ, 04CR0ZZ, 04CR4ZZ, 04CS0ZZ, 04CS4ZZ, 04CT0ZZ, 04CT4ZZ, 04CU0ZZ, 04CU4ZZ, 04CV0ZZ, 04CV4ZZ, 04CW0ZZ, 04CW4ZZ, 04CY0ZZ, 04CY4ZZ, 04HY02Z, 04HY42Z, 04PY0YZ, 04PY3YZ, 04PY4YZ, 04WY0YZ, 04WY3YZ, 04WY4YZ, 04BK0ZZ, 04BK4ZZ, 04BL0ZZ, 04BL4ZZ, 04BM0ZZ, 04BM4ZZ, 04BN0ZZ, 04BN4ZZ, 04BP0ZZ, 04BP4ZZ, 04BQ0ZZ, 04BQ477, 04BR0ZZ, 04BR4ZZ, 04BS0ZZ, 04BS4ZZ, 04BT0ZZ, 04BT4ZZ, 04BU0ZZ, 04BU4ZZ, 04BV0ZZ, 04BV4ZZ, 04BW0ZZ, 04BW4ZZ, 04BY0ZZ, 04BY4ZZ, 04RK07Z, 04RK0JZ, 04RK0KZ, 04RK47Z, 04RK4JZ, 04RK4KZ, 04RL07Z, 04RL0JZ, 04RL0KZ, 04RL47Z, 04RL4JZ, 04RL4KZ, 04RM07Z, 04RM0JZ, 04RM0KZ, 04RM47Z, 04RM4JZ, 04RM4KZ, 04RN07Z, 04RN0JZ, 04RN0KZ, 04RN47Z, 04RN4JZ, 04RN4KZ, 04RP07Z, 04RP0JZ, 04RP0KZ, 04RP47Z,

© 2022 Aday AW et al. *JAMA Network Open.*

Description	ICD-9 Procedure Codes	CPT Codes	ICD-10 Procedure Codes
			04RP4JZ, 04RP4KZ, 04RQ07Z, 04RQ0JZ, 04RQ0KZ, 04RQ47Z, 04RQ4JZ, 04RQ4KZ, 04RR07Z, 04RR0JZ, 04RR0KZ, 04RR47Z, 04RR4JZ, 04RR4KZ, 04RS07Z, 04RS0JZ, 04RS0KZ, 04RS47Z, 04RS4JZ, 04RS4KZ, 04RT07Z, 04RT0JZ, 04RT0KZ, 04RT47Z, 04RT4JZ, 04RT4KZ, 04RU07Z, 04RU0JZ, 04RU0KZ, 04RU47Z, 04RU4JZ, 04RU4KZ, 04RV07Z, 04RV0JZ, 04RV0KZ, 04RV47Z, 04RV4JZ, 04RV4KZ, 04RW07Z, 04RW0JZ, 04RW0KZ, 04RW47Z, 04RW4JZ, 04RW4KZ, 04RY07Z, 04RY0JZ, 04RY0KZ, 04RY47Z, 04RY4JZ, 04RY4KZ, 03CY0ZZ, 03CY3ZZ, 03CY4ZZ, 03QY0ZZ, 03QY3ZZ, 03QY4ZZ, 04CY0ZZ, 04CY3ZZ, 04CY4ZZ, 04QY0ZZ, 04QY3ZZ, 04QY4ZZ, 05CY0ZZ, 05CY3ZZ, 05CY4ZZ, 05QY0ZZ, 05QY3ZZ, 05QY4ZZ, 06CY0ZZ, 06CY3ZZ, 06CY4ZZ, 06QY0ZZ, 06QY3ZZ, 06QY4ZZ, 04UC07Z, 04UC37Z, 04UC47Z, 04UD07Z, 04UD37Z, 04UD47Z, 04UE07Z, 04UE37Z, 04UE47Z, 04UF07Z, 04UF37Z, 04UF47Z, 04UH07Z, 04UH37Z, 04UH47Z, 04UJ07Z, 04UJ37Z, 04UJ47Z, 04UK07Z, 04UK37Z, 04UK47Z, 04UL07Z, 04UL37Z, 04UL47Z, 04UM07Z, 04UM37Z, 04UM47Z, 04UN07Z, 04UN37Z, 04UN47Z, 04UP07Z, 04UP37Z, 04UP47Z, 04UQ07Z, 04UQ37Z, 04UQ47Z, 04UR07Z, 04UR37Z, 04UR47Z, 04US07Z, 04US37Z, 04US47Z, 04UT07Z, 04UT37Z, 04UT47Z, 04UU07Z, 04UU37Z, 04UU47Z, 04UV07Z, 04UV37Z, 04UV47Z, 04UW07Z, 04UW37Z, 04UW47Z, 04UY07Z, 04UY37Z, 04UY47Z, 04UC0JZ, 04UC3JZ, 04UC4JZ, 04UD0JZ, 04UD3JZ, 04UD4JZ, 04UE0JZ, 04UE3JZ, 04UE4JZ, 04UF0JZ, 04UF3JZ, 04UF4JZ, 04UH0JZ, 04UH3JZ, 04UH4JZ, 04UJ0JZ, 04UJ3JZ, 04UJ4JZ, 04UK0JZ, 04UK3JZ, 04UK4JZ, 04UL0JZ, 04UL3JZ, 04UL4JZ, 04UM0JZ, 04UM3JZ, 04UM4JZ, 04UN0JZ, 04UN3JZ, 04UN4JZ, 04UP0JZ, 04UP3JZ, 04UP4JZ, 04UQ0JZ, 04UQ3JZ, 04UQ4JZ, 04UR0JZ, 04UR3JZ, 04UR4JZ, 04US0JZ, 04US3JZ, 04US4JZ, 04UT0JZ, 04UT3JZ, 04UT4JZ, 04UU0JZ, 04UU3JZ, 04UU4JZ, 04UV0JZ, 04UV3JZ, 04UV4JZ, 04UW0JZ, 04UW3JZ, 04UW4JZ, 04UY0JZ, 04UY3JZ, 04UY4JZ, 04UC0KZ, 04UC3KZ, 04UC4KZ, 04UD0KZ, 04UD3KZ, 04UD4KZ, 04UE0KZ, 04UE3KZ, 04UE4KZ, 04UF0KZ, 04UF3KZ, 04UF4KZ, 04UH0KZ, 04UH3KZ, 04UH4KZ, 04UJ0KZ, 04UJ3KZ, 04UJ4KZ, 04UK0KZ, 04UK3KZ, 04UK4KZ, 04UL0KZ, 04UL3KZ, 04UL4KZ, 04UM0KZ, 04UM3KZ, 04UM4KZ, 04UN0KZ, 04UN3KZ, 04UN4KZ, 04UP0KZ, 04UP3KZ, 04UP4KZ, 04UQ0KZ, 04UQ3KZ, 04UQ4KZ, 04UR0KZ, 04UR3KZ, 04UR4KZ, 04US0KZ, 04US3KZ, 04US4KZ, 04UT0KZ, 04UT3KZ, 04UT4KZ, 04UU0KZ, 04UU3KZ, 04UU4KZ, 04UV0KZ, 04UV3KZ, 04UV4KZ, 04UW0KZ, 04UW3KZ, 04UW4KZ, 04UY0KZ, 04UY3KZ, 04UY4KZ
Non-Coronary Vessel Percutaneous Transluminal Mechanical Thrombectomy, or Stents	39.90	37184, 37185, 37186, 37205, 37206, 37207, 37208	--

Prevalent and incident peripheral artery disease was defined by the presence of these Veterans Health Administration, Veterans Health Administration fee-for-service, or Medicare diagnostic and procedural codes in addition to qualifying ankle-brachial indices.
CPT, current procedural terminology; ICD, International Classification of Disease.

© 2022 Aday AW et al. *JAMA Network Open.*

**eTable 2.** Baseline Characteristics of Individuals Excluded From the Study					
	Patients, No. (%)				
Characteristic	Men (n = 170,752)[a]	Women (n = 5,430)[b]	Black (n = 35,603)[c]	White (n = 122,631)[d]	Other (n = 9,598)[e]
Age, mean (SD), y	61.1 (6.3)	57.8 (6.6)	59.9 (6.4)	61.3 (6.2)	60.9 (6.3)
Male	NA	NA	33,998 (95.5)	119,306 (97.3)	9,300 (96.9)
Race/ethnicity					
Black	33,998 (20.9)	1,605 (30.7)	NA	NA	NA
White	119,306 (73.4)	3,325 (63.6)	NA	NA	NA
Other[f]	9,300 (5.7)	298 (5.7)	NA	NA	NA
Smoking status					
Current	93,800 (56.5)	2,594 (49.0)	19,516 (55.8)	68,462 (57.2)	4,193 (45.2)
Former	41,865 (25.2)	1,007 (19.0)	7,410 (21.2)	31,179 (26.0)	2,599 (28.0)
Never	30,208 (18.2)	1,698 (32.0)	8,027 (23.0)	20,112 (16.8)	2,491 (26.8)
Diabetes	90,255 (52.9)	2,244 (41.3)	20,351 (57.2)	62,190 (50.7)	6,170 (64.3)
Prevalent CVD	87,220 (51.1)	1,862 (34.3)	16,663 (46.8)	63,744 (52.0)	4,841 (50.4)
Left ankle-brachial index, median (Q1, Q3)	0.9 (0.6, 1.0)	0.9 (0.7, 1.0)	0.8 (0.6, 1.0)	0.9 (0.6, 1.0)	0.9 (0.7, 1.1)
Right ankle-brachial index, median (Q1, Q3)	0.9 (0.7, 1.0)	1.0 (0.7, 1.0)	0.9 (0.7, 1.0)	0.9 (0.7, 1.0)	1.0 (0.7, 1.1)
SBP, mean (SD), mmHg	131.6 (14.3)	129.7 (14.8)	133.3 (14.7)	131.0 (14.1)	131.1 (14.1)
DBP, mean (SD), mmHg	75.8 (8.7)	75.2 (8.8)	77.7 (8.8)	75.2 (8.6)	75.2 (8.7)
Antihypertensive therapy	164,191 (96.2)	5,021 (92.5)	34,454 (96.8)	118,278 (96.5)	9,264 (96.5)
BMI, mean (SD), kg/m^2	29.6 (6.6)	30.3 (7.4)	28.8 (6.4)	29.9 (6.7)	30.1 (6.5)
Total cholesterol, median (Q1, Q3), mg/dL	169 (142, 201)	192 (164, 224)	171 (143, 202)	169 (142, 201)	167 (139, 199)
LDL cholesterol, median (Q1, Q3), mg/dL	95 (72, 122)	109 (86, 138)	97 (74, 125)	95 (72, 122)	92 (70, 120)
HDL cholesterol, median (Q1, Q3), mg/dL	39 (33, 48)	49 (40, 60)	43 (35, 53)	39 (32, 47)	39 (33, 47)
Triglycerides, median (Q1, Q3), mg/dL	140 (95, 212)	134 (92, 203)	115 (80, 169)	147 (100, 223)	145 (99, 218)
Statin therapy	144,879 (84.9)	4,130 (76.1)	29,324 (82.4)	105,647 (86.2)	8,199 (85.4)
eGFR, mean (SD), mL/min/1.73m^2	82.6 (29.4)	80.8 (27.1)	85.0 (33.4)	82.2 (27.3)	79.7 (35.9)

SI conversion factors: To convert LDL and HDL cholesterol to mmol/L, multiply values by 0.0259. To convert triglycerides to mmol/L, multiply by 0.0113.

BMI, body-mass index; CVD, cardiovascular disease; DBP, diastolic blood pressure; eGFR, estimated glomerular filtration rate; HDL, high-density lipoprotein; LDL, low-density lipoprotein; PAD, peripheral artery disease; SBP, systolic blood pressure; SD, standard deviation.

Other race and ethnicity included individuals identified as American Indian or Alaska Native, Asian, or Native Hawaiian or other Pacific Islander, as well as those with Hispanic ethnicity.

a Number missing: 14 (Age); 8,148 (Race/ethnicity); 4,879 (Smoking Status); 88,148 (Left ABI); 80,783 (Right ABI); 5,042 (SBP); 5,042 (DBP); 2,873 (BMI); 6,939 (Total cholesterol); 8,738 (LDL cholesterol); 7,806 (HDL cholesterol); 8,010 (Triglycerides); 2,888 (eGFR)

b Number missing: 1 (Age); 202 (Race/ethnicity); 131 (Smoking Status); 2,921 (Left ABI); 2,591 (Right ABI); 561 (SBP); 561 (DBP); 66 (BMI); 177 (Total cholesterol); 231 (LDL cholesterol); 203 (HDL cholesterol); 217 (Triglycerides); 99 (eGFR)

c Number missing: 6 (Age); 650 (Smoking Status); 18,527 (Left ABI); 16,611 (Right ABI); 1,166 (SBP); 1,166 (DBP); 454 (BMI); 1,319 (Total Cholesterol); 1,736 (LDL Cholesterol); 1,530 (HDL Cholesterol); 1,583 (Triglycerides); 428 (eGFR)

d Number missing: 5 (Age); 2,878 (Smoking Status); 63,579 (Left ABI); 57,979 (Right ABI); 3,096 (SBP); 3,096 (DBP); 1,582 (BMI); 4,259 (Total Cholesterol); 5,365 (LDL Cholesterol); 4,744 (HDL Cholesterol); 4,900 (Triglycerides); 1,740 (eGFR)

e Number missing: 2 (Age); 315 (Smoking Status); 4,771 (Left ABI); 4,803 (Right ABI); 268 (SBP); 268 (DBP); 112 (BMI); 284 (Total Cholesterol); 378 (LDL Cholesterol); 349 (HDL Cholesterol); 346 (Triglycerides); 132 (eGFR)

f Other race and ethnicity included individuals identified as American Indian or Alaska Native, Asian, or Native Hawaiian or other Pacific Islander, as well as those with Hispanic ethnicity.

eTable 3. Procedural Codes for Lower-Extremity Amputation Due to Trauma or Non–Peripheral Artery Disease Indications

Description	ICD-9 Codes	ICD-10 Codes
Lower Extremity Trauma/Injury and Others	736.30, 736.31, 736.32, 736.39, 736.4, 736.41, 736.42, 736.5, 736.6, 736.70, 736.71, 736.72, 736.73, 736.74, 736.75, 736.76, 736.79, 736.81, 736.89, 736.9, 735.0, 735.1, 735.2, 735.3, 735.4, 735.5, 735.8, 735.9, 754.30, 754.31, 754.32, 754.33, 754.35, 754.40, 754.41, 754.42, 754.43, 754.44, 754.50, 754.51, 754.52, 754.53, 754.59, 754.60, 754.61, 754.62, 754.69, 754.70, 754.71, 754.79, 755.01, 755.13, 755.14, 755.30, 755.31, 755.32, 755.33, 755.34, 755.35, 755.36, 755.37, 755.38, 755.39, 755.4, 755.60, 755.61, 755.62, 755.63, 755.64, 755.65, 755.66, 755.67, 755.69, 755.8, 759.7, 759.89, 890.0, 890.1, 890.2, 891.0, 891.1, 891.2, 895.0, 895.1, 896.0, 896.1, 896.2, 896.3, 897.0, 897.1, 897.2, 897.3, 897.4, 897.5, 897.6, 897.7, 820.00, 820.01, 820.02, 820.03, 820.09, 820.10, 820.11, 820.12, 820.13, 820.19, 820.20, 820.21, 820.22, 820.30, 820.31, 820.32, 820.8, 820.9, 821.00, 821.01, 821.10, 821.11, 821.20, 821.21, 821.22, 821.23, 821.29, 821.30, 821.31, 821.32, 821.33, 821.39, 822.0, 822.1, 823.00, 823.01, 823.02, 823.10, 823.11, 823.12, 823.20, 823.21, 823.22, 823.30, 823.31, 823.32, 823.40, 823.41, 823.42, 823.80, 823.81, 823.82, 823.90, 823.91, 823.92, 824.0, 824.1, 824.2, 824.3, 824.4, 824.5, 824.6, 824.7, 824.8, 824.9, 825.0, 825.1, 825.20, 825.21, 825.22, 825.23, 825.24, 825.25, 825.29, 825.30, 825.31, 825.32, 825.33, 825.34, 825.35, 825.39, 826.0, 826.1, 827.0, 827.1, 828.0, 828.1, 829.0, 829.1, 835.00, 835.01, 835.02, 835.03, 835.10, 835.11, 835.12, 835.13, 836.0, 836.1, 836.2, 836.3, 836.4, 836.50, 836.51, 836.52, 836.53, 836.54, 836.59, 836.60, 836.61, 836.62, 836.63, 836.64, 836.69, 837.0, 837.1, 838.00, 838.01, 838.02, 838.03, 838.04, 838.05, 838.06, 838.09, 838.10, 838.11, 838.12, 838.13, 838.14, 838.15, 838.16, 838.19, 904.0, 904.1, 904.2, 904.3, 904.40, 904.41, 904.42, 904.50, 904.51, 904.52, 904.53, 904.54, 904.6, 904.7, 904.8, 904.9, 928.00, 928.01, 928.10, 928.11, 928.20, 928.21, 928.3, 928.8, 928.9, 929.0, 929.9, 959.6, 959.7, 996.40, 996.41, 996.42, 996.43, 996.44, 996.45, 996.46, 996.47, 996.49, 996.66, 996.67, 996.78	M21.959, M21.059, M21.159, M21.859, M21.069, M21.169, M21.869, M21.869, M21.969, M21.549, M21.6X9, M21.6X9, M21.539, M21.6X9, M21.6X9, M21.6X9, M21.769, M21.759, M21.969, M21.80, M21.90, M20.10, M20.30, M20.20, M20.40, M20.40, M20.5X9, M20.5X9, M20.60, Q65.00, Q65.1, Q65.30, Q65.4, Q65.01, Q65.32, Q65.02, Q65.31, Q68.2, Q68.2, Q68.3, Q68.4, Q68.5, Q66.0, Q66.0, Q66.21, Q66.22, Q66.1, Q66.3, Q66.4, Q66.50, Q66.80, Q66.4, Q66.6, Q66.89, Q66.7, Q66.89, Q69.0, Q69.1, Q70.30, Q70.20, Q72.899, Q72.00, Q72.899, Q72.10, Q72.40, Q72.20, Q72.50, Q72.60, Q72.30, Q72.70, Q72.30, Q72.70, Q73.0, Q73.1, Q73.8, Q74.2, Q65.81, Q65.82, Q65.89, Q68.2, Q74.1, Q74.2, Q66.89, Q66.89, Q74.2, Q74.8, Q89.7, E78.71, E78.72, Q87.2, Q87.3, Q87.5, Q87.81, Q87.82, Q87.89, Q89.8, S71.009A, S71.109A, S71.029A, S71.129A, S71.009A, S71.109A, S76.929A, S81.009A, S81.809A, S91.009A, S81.029A, S81.829A, S91.029A, S81.009A, S81.809A, S86.929A, S91.009A, S96.929A, S98.119A, S98.129A, S98.139A, S98.149A, S98.219A, S98.229A, S98.119A, S98.129A, S98.139A, S98.149A, S98.219A, S98.229A, S98.019A, S98.029A, S98.319A, S98.329A, S98.919A, S98.929A, S98.019A, S98.029A, S98.319A, S98.329A, S98.919A, S98.929A, S98.911A, S98.921A, S98.912A, S98.922A, S98.911A, S98.912A, S88.119A, S88.129A, S88.119A, S88.129A, S78.019A, S78.029A, S78.119A, S78.129A, S78.919A, S78.929A, S88.019A, S88.029A, S78.019A, S78.029A, S78.119A, S78.129A, S88.019A, S88.029A, S78.919A, S78.929A, S88.919A, S88.929A, S78.919A, S78.929A, S88.919A, S88.929A, S88.911A, S88.912A, S88.911A, S88.912A, S72.019A, S72.023A, S72.026A, S72.033A, S72.036A, S72.043A, S72.046A, S72.099A, S72.019B, S72.019C, S72.023B, S72.023C, S72.026B, S72.026C, S72.033B, S72.033C, S72.036B, S72.036C, S72.043B, S72.043C, S72.046B, S72.046C, X72.099B, S72.099C, S72.109A, S72.143A, S72.146A, S72.23XA, S72.26XA, S72.109B, S72.109C, S72.143B, S72.143C, S72.146B, S72.146C, S72.23XB, S72.23XC, S72.26XB, S72.26XC, S72.009A, S72.009B,

© 2022 Aday AW et al. *JAMA Network Open.*

Description	ICD-9 Codes	ICD-10 Codes
		S72.009C, S72.90XA, S72.309A, S72.90XB, S72.90XC, S72.309B, S72.309C, S72.409A, S72.413A, S72.416A, S72.443A, S72.446A, S72.453A, S72.456A, S72.499A, S72.409B, S72.409C, S72.413B, S72.413C, S72.416B, S72.416C, S72.443B, S72.443C, S72.446B, S72.446C, S72.453B, S72.453C, S72.456B, S72.456C, S72.499B, S72.499C, S82.009A, S82.009B, S82.009C, S82.109A, S82.839A, S82.101A, S82.831A, S82.102A, S82.832A, S82.109B, S82.109C, S82.839B, S82.839C, S82.101B, S82.831B, S82.102B, S82.832B, S82.209A, S82.409A, S82.201A, S82.401A, S82.202A, S82.402A, S82.209B, S82.209C, S82.409B, S82.409C, S82.201B, S82.401B, S82.202B, S82.402B, S82.169A, S82.319A, S82.819A, S82.829A, 0LB00ZZ, 0LB03ZZ, 0LB04ZZ, 0LB10ZZ, 0LB13ZZ, 0LB14ZZ, 0LB20ZZ, 0LB23ZZ, 0LB24ZZ, 0LB30ZZ, 0LB33ZZ, 0LB34ZZ, 0LB40ZZ, 0LB43ZZ, 0LB44ZZ, 0LB50ZZ, 0LB53ZZ, 0LB54ZZ, 0LB60ZZ, 0LB63ZZ, 0LB64ZZ, 0LB90ZZ, 0LB93ZZ, 0LB94ZZ, 0LBB0ZZ, 0LBB3ZZ, 0LBB4ZZ, 0LBC0ZZ, 0LBC3ZZ, 0LBC4ZZ, 0LBD0ZZ, 0LBD3ZZ, 0LBD4ZZ, 0LBF0ZZ, 0LBF3ZZ, 0LBF4ZZ, 0LBG0ZZ, 0LBG3ZZ, 0LBG4ZZ, 0LBH0ZZ, 0LBH3ZZ, 0LBH4ZZ, 0LBJ0ZZ, 0LBJ3ZZ, 0LBJ4ZZ, 0LBK0ZZ, 0LBK3ZZ, 0LBK4ZZ, 0LBL0ZZ, 0LBL3ZZ, 0LBL4ZZ, 0LBM0ZZ, 0LBM3ZZ, 0LBM4ZZ, 0LBN0ZZ, 0LBN3ZZ, 0LBN4ZZ, 0LBP0ZZ, 0LBP3ZZ, 0LBP4ZZ, 0LBQ0ZZ, 0LBQ3ZZ, 0LBQ4ZZ, 0LBR0ZZ, 0LBR3ZZ, 0LBR4ZZ, 0LBS0ZZ, 0LBS3ZZ, 0LBS4ZZ, 0LBT0ZZ, 0LBT3ZZ, 0LBT4ZZ, 0LBV0ZZ, 0LBV3ZZ, 0LBV4ZZ, 0LBW0ZZ, 0LBW3ZZ, 0LBW4ZZ, 0LT00ZZ, 0LT04ZZ, 0LT10ZZ, 0LT14ZZ, 0LT20ZZ, 0LT24ZZ, 0LT30ZZ, 0LT34ZZ, 0LT40ZZ, 0LT44ZZ, 0LT50ZZ, 0LT54ZZ, 0LT60ZZ, 0LT64ZZ, 0LT00ZZ, 0LT94ZZ, 0LTB0ZZ, 0LTB4ZZ, 0LTC0ZZ, 0LTC4ZZ, 0LTD0ZZ, 0LTD4ZZ, 0LTF0ZZ, 0LTF4ZZ, 0LTG0ZZ, 0LTG4ZZ, 0LTH0ZZ, 0LTH4ZZ, 0LTJ0ZZ, 0LTJ4ZZ, 0LTK0ZZ, 0LTK4ZZ, 0LTL0ZZ, 0LTL4ZZ, 0LTM0ZZ, 0LTM4ZZ, 0LTN0ZZ, 0LTN4ZZ, 0LTP0ZZ, 0LTP4ZZ, 0LTQ0ZZ, 0LTQ4ZZ, 0LTR0ZZ, 0LTR4ZZ, 0LTS0ZZ, 0LTS4ZZ, 0LTT0ZZ, 0LTT4ZZ, 0LTV0ZZ, 0LTV4ZZ, 0LTW0ZZ, 0LTW4ZZ, S82.201A, S82.401A, S82.201A, S82.401A, S82.202A, S82.402A, S82.201B, S82.201C, S82.401B, S82.401C, S82.201B, S82.401B, S82.202B, S82.402B, S82.53XA, S82.56XA, S82.53XB, S82.53XC, S82.56XB, S82.56XC, S82.63XA,

© 2022 Aday AW et al. *JAMA Network Open.*

Description	ICD-9 Codes	ICD-10 Codes
		S82.66XA, S82.63XB, S82.63XC, S82.66XB, S82.66XC, S82.843A, S82.846A, S82.843B, S82.843C, S82.846B, S82.846C, S82.853A, S82.856A, S82.853B, S82.853C, S82.856B, S82.856C, S82.899A, S82.899B, S82.899C, S92.009A, S99.009A, S99.019A, S99.029A, S99.039A, S99.049A, S99.099A, S92.009B, S99.009B, S99.019B, S99.029B, S99.039B, S99.049B, S99.099B, S92.819A, S92.909A, S92.109A, S92.253A, S92.256A, S92.213A, S92.216A, S92.223A, S92.226A, S92.233A, S92.236A, S92.243A, S92.246A, S92.309A, S99.109A, S99.119A, S99.129A, S99.139A, S99.149A, S99.199A, S92.201A, S92.202A, S92.209A, S92.819B, S92.909B, S92.109B, S92.253B, S92.256B, S92.213B, S92.216B, S92.223B, S92.226B, S92.233B, S92.236B, S92.243B, S92.246B, S92.309B, S99.109B, S99.119B, S99.129B, S99.139B, S99.149B, S99.199B, S92.201B, S92.202B, S92.209B, S92.403A, S92.406A, S92.503A, S92.506A, S92.403B, S92.406B, S92.503B, S92.506B, S82.90XA, S82.90XB, S72.91XA, S82.91XA, S72.92XA, S82.92XA, S42.90XA, S52.90XA, S72.90XA, S82.90XA, S22.20XA, S22.49XA, S72.90XA, S82.90XA, T14.8XXA, T14.8XXA, T14.8XXA, S73.006A, S73.016A, S73.026A, S73.036A, S71.009A, S73.016A, S71.009A, S73.026A, S71.009A, S73.036A, S83.219A, S83.249A, S83.289A, S83.209A, S83.30XA, S83.006A, S81.009A, S83.006A, S83.106A, S83.116A, S83.126A, S83.136A, S83.146A, S83.196A, S81.009A, S83.106A, S81.009A, S83.116A, S81.009A, S83.126A, S81.009A, S83.136A, S81.009A, S83.146A, S81.009A, S83.196A, S93.06XA, S91.009A, S93.06XA, S93.306A, S93.316A, S93.316A, S93.326A, S93.336A, S93.129A, S93.119A, S93.106A, S93.336A, S91.309A, S93.306A, S91.309A, S93.316A, S91.309A, S93.316A, S91.309A, S93.326A, S91.309A, S93.336A, S91.309A, S93.129A, S91.109A, S93.119A, S91.109A, S93.336A, S75.009A, S75.009A, S75.109A, S75.209A, S85.309A, S85.409A, S85.009A, S85.509A, S85.009A, S85.509A, S85.109A, S85.139A, S85.809A, S85.169A, S85.809A, S95.109A, S75.809A, S85.809A, S95.809A, S75.909A, S85.909A, S95.902A, T14.90XA, S77.10XA, S77.00XA, S87.80XA,

© 2022 Aday AW et al. *JAMA Network Open.*

Description	ICD-9 Codes	ICD-10 Codes
		S87.00XA, S97.80XA, S97.00XA, S97.109A, S77.20XA, S77.20XA, S77.20XA, S77.20XA, S79.819A, S79.829A, S79.919A, S79.929A, S89.80XA, S89.90XA, S99.819A, S99.919A, T84.498A, T84.039A, T84.029A, T84.019A, M97.9XXA, T84.059A, T84.069A, T84.099A, T84.119A, T84.129A, T84.199A, T84.498A, T84.50XA, T84.60XA, T84.7XXA, T84.81XA, T84.82XA, T84.83XA, T84.84XA, T84.85XA, T84.86XA, T84.89XA, T84.9XXA
Amputation with Non-PAD Indications	170.6, 170.7, 170.8, 170.9, 171.3, 172.7, 173.70, 173.71, 173.72, 173.79, 198.5, 344.1, 711.00, 711.01, 711.02, 711.03, 711.04, 711.05, 711.06, 711.07, 711.08, 711.09, 728.6, 733.20, 733.21, 733.22, 733.29, 736.30, 736.31, 736.32, 736.39, 736.41, 736.42, 736.5, 736.6, 736.70, 736.71, 736.72, 736.73, 736.74, 736.75, 736.76, 736.79, 736.81, 736.89, 736.9, 735.0, 735.1, 735.2, 735.3, 735.4, 735.5, 735.8, 735.9, 754.30, 754.31, 754.32, 754.33, 754.35, 754.40, 754.41, 754.42, 754.43, 754.44, 754.50, 754.51, 754.52, 754.53, 754.59, 754.60, 754.61, 754.62, 754.69, 754.70, 754.71, 754.79, 755.02, 755.13, 755.14, 755.30, 755.31, 755.32, 755.33, 755.34, 755.35, 755.36, 755.37, 755.38, 755.39, 755.4, 755.60, 755.61, 755.62, 755.63, 755.64, 755.65, 755.66, 755.67, 755.69, 755.8, 759.7, 759.89, 890.0, 890.1, 890.2, 891.0, 891.1, 891.2, 895.0, 895.1, 896.0, 896.1, 896.2, 896.3, 897.0, 897.1, 897.2, 897.3, 897.4, 897.5, 897.6, 897.7, 820.00, 820.01, 820.02, 820.03, 820.09, 820.10, 820.11, 820.12, 820.13, 820.19, 820.20, 820.21, 820.22, 820.30, 820.31, 820.32, 820.8, 820.9, 821.00, 821.01, 821.10, 821.11, 821.20, 821.21, 821.22, 821.23, 821.29, 821.30, 821.31, 821.32, 821.33, 821.39, 822.0, 822.1, 823.00, 823.01, 823.02, 823.10, 823.11, 823.12, 823.20, 823.21, 823.22, 823.30, 823.31, 823.32, 823.40, 823.41, 823.42, 823.80, 823.81, 823.82, 823.90, 823.91, 823.92, 824.0, 824.1, 824.2, 824.3, 824.4, 824.5, 824.6, 824.7, 824.8, 824.9, 825.0, 825.1, 825.20, 825.21, 825.22, 825.23, 825.24, 825.25, 825.29, 825.30, 825.31, 825.32, 825.33, 825.34, 825.35, 825.39, 826.0, 826.1, 827.0, 827.1, 828.0, 828.1, 829.0, 829.1, 835.00, 835.01, 835.02, 835.03, 835.10, 835.11, 835.12, 835.13, 836.0, 836.1, 836.2, 836.3, 836.4, 836.50, 836.51, 836.52, 836.53, 836.54, 836.59, 836.60, 836.61, 836.62, 836.63, 836.64, 836.69, 837.0, 837.1, 838.00, 838.01, 838.02, 838.03, 838.04, 838.05, 838.06, 838.09, 838.10, 838.11, 838.12, 838.13, 838.14, 838.15, 838.16, 838.19,	C41.4, C49.20, C40.20, C40.30, C41.9, C43.70, D03.70, D03.71, D03.72, C44.701, C44.711, C44.721, C44.791, C79.51, C79.52, G82.20, M00.00, M00.10, M00.20, M00.80, M00.9, M00.019, M00.119, M00.219, M00.819, M00.029, M00.129, M00.229, M00.829, M00.039, M00.139, M00.239, M00.839, M00.049, M00.149, M00.249, M00.849, M00.059, M00.159, M00.259, M00.859, M00.069, M00.169, M00.269, M00.869, M00.079, M00.179, M00.279, M00.879, M00.08, M00.18, M00.28, M00.88, M00.9, M00.09, M00.19, M00.29, M00.89, M72.0, M85.60, M85.40, M85.50, M85.00, M85.60, M21.959, M85.40, M85.50, M85.00, M85.60, M21.959, M21.059, M21.159, M21.859, M21.069, M21.169, M21.869, M21.869, M21.969, M21.549, M21.6X9, M21.6X9, M21.539, M21.6X9, M21.6X9, M21.6X9, M21.759, M21.769, M21.80, M21.969, M21.90, M20.10, M20.30, M20.20, M20.40, M20.40, M20.5X9, M20.5X9, M20.60, Q65.00, Q65.1, Q65.30, Q65.4, Q68.2, Q68.2, Q68.2, Q68.3, Q68.4, Q68.5, Q66.0, Q66.0, Q66.21, Q66.22, Q66.1, Q66.3, Q66.4, Q66.50, Q66.80, Q66.4, Q66.6, Q67.0, Q67.1, Q67.2, Q67.3, Q67.4, Q66.7, Q66.89, Q69.0, Q69.1, Q70.30, Q70.20, Q72.899, Q72.00, Q72.899, Q72.10, Q72.40, Q72.20, Q72.50, Q72.60, Q72.30, Q72.70, Q72.30, Q72.70, Q73.0, Q73.1, Q73.8, Q74.2, Q65.81, Q65.82, Q65.89, Q68.2, Q74.1, Q74.2, Q66.89, Q66.89, Q74.2, Q74.8, Q89.7, E78.71, E78.72, Q87.2, Q87.3, Q87.5, Q87.81, Q87.82, Q87.89, Q89.8, S71.009A, S71.109A, S71.029A, S71.129A, S71.009A, S71.109A, S76.929A, S81.009A, S81.809A, S91.009A, S81.029A, S81.829A, S91.029A, S81.009A, S81.809A, S86.929A, S91.009A, S96.929A, S98.119A, S98.129A, S98.139A, S98.149A, S98.219A, S98.229A, S98.119A, S98.129A, S98.139A, S98.149A, S98.219A, S98.229A, S98.019A, S98.029A, S98.319A, S98.329A, S98.919A, S98.929A, S98.019A,

© 2022 Aday AW et al. *JAMA Network Open.*

Description	ICD-9 Codes	ICD-10 Codes
	904.0, 904.1, 904.2, 904.3, 904.40, 904.41, 904.42, 904.50, 904.51, 904.52, 904.53, 904.54, 904.6, 904.7, 904.8, 904.9, 905.4, 928.00, 928.01, 928.10, 928.11, 928.20, 928.21, 928.3, 928.8, 928.9, 929.0, 929.9, 959.6, 959.7, 996.40, 996.41, 996.42, 996.43, 996.44, 996.45, 996.46, 996.47, 996.49, 996.66, 996.67, 996.78	S98.029A, S98.319A, S98.329A, S98.919A, S98.929A, S98.911A, S98.921A, S98.912A, S98.922A, S98.911A, S98.912A, S88.119A, S88.129A, S88.119A, S88.129A, S78.019A, S78.029A, S78.119A, S78.129A, S78.919A, S78.929A, S88.019A, S88.029A, S78.019A, S78.029A, S78.119A, S78.129A, S88.019A, S88.029A, S78.919A, S78.929A, S88.919A, S88.929A, S78.919A, S78.929A, S88.919A, S88.929A, S88.911A, S88.912A, S88.911A, S88.912A, S72.019A, S72.023A, S72.026A, S72.033A, S72.036A, S72.043A, S72.046A, S72.099A, S72.019B, S72.019C, S72.023B, S72.023C, S72.026B, S72.026C, S72.033B, S72.033C, S72.036B, S72.036C, S72.043B, S72.043C, S72.046B, S72.046C, S72.099B, S72.099C, S72.109A, S72.143A, S72.146A, S72.23XA, S72.26XA, S72.109B, S72.109C, S72.143B, S72.143C, S72.146B, S72.146C, S72.23XB, S72.23XC, S72.26XB, S72.26XC, S72.009A, S72.009B, S72.009C, S72.90XA, S72.309A, S72.90XB, S72.90XC, S72.309B, S72.309C, S72.409A, S72.413A, S72.416A, S72.443A, S72.446A, S72.453A, S72.456A, S72.499A, S72.409B, S72.409C, S72.413B, S72.413C, S72.416B, S72.416C, S72.443B, S72.443C, S72.446B, S72.446C, S72.453B, S72.453C, S72.456B, S72.456C, S72.499B, S72.499C, S82.009A, S82.009B, S82.009C, S82.109A, S82.839A, S82.101A, S82.831A, S82.102A, S82.832A, S82.109B, S82.109C, S82.839B, S82.839C, S82.101B, S82.831B, S82.102B, S82.832B, S82.209A, S82.409A, S82.201A, S82.401A, S82.202A, S82.402A, S82.209B, S82.209C, S82.409B, S82.409C, S82.201B, S82.401B, S82.202B, S82.402B, S82.169A, S82.319A, S82.201A, S82.401A, S82.202A, S82.402A, S82.819A, S82.829A, S82.161A, S82.811A, S82.311A, S82.821A, S82.162A, S82.812A, S82.312A, S82.822A, S82.401A, S82.201A, S82.401A, S82.202A, S82.402A, S82.201B, S82.201C, S82.401B, S82.401C, S82.201B, S82.401B, S82.202B, S82.402B, S82.53XA, S82.56XA, S82.53XB, S82.53XC, S82.56XB, S82.56XC, S82.63XA, S82.66XA, S82.63XB, S82.63XC, S82.66XB, S82.66XC, S82.843A, S82.846A, S82.843B, S82.843C, S82.846B, S82.846C, S82.853A, S82.856A, S82.853B, S82.853C,

© 2022 Aday AW et al. *JAMA Network Open.*

Description	ICD-9 Codes	ICD-10 Codes
		S82.856B, S82.856C, S82.899A, S82.899B, S82.899C, S92.009A, S99.009A, S99.019A, S99.029A, S99.039A, S99.049A, S99.099A, S92.009B, S99.009B, S99.019B, S99.029B, S99.039B, S99.049B, S99.099B, S92.819A, S92.909A, S92.109A, S92.253A, S92.256A, S92.213A, S92.216A, S92.223A, S92.226A, S92.233A, S92.236A, S92.243A, S92.246A, S92.309A, S99.109A, S99.119A, S99.129A, S99.139A, S99.149A, S99.199A, S92.201A, S92.202A, S92.209A, S92.819B, S92.909B, S92.109B, S92.253B, S92.256B, S92.213B, S92.216B, S92.223B, S92.226B, S92.233B, S92.236B, S92.243B, S92.246B, S92.309B, S99.109B, S99.119B, S99.129B, S99.139B, S99.149B, S99.199B, S92.201B, S92.202B, S92.209B, S92.403A, S92.406A, S92.503A, S92.506A, S92.403B, S92.406B, S92.503B, S92.506B, S82.90XA, S72.91XA, S82.91XA, S72.92XA, S82.92XA, S42.90XA, S52.90XA, S72.90XA, S82.90XA, S22.20XA, S22.49XA, S72.90XA, S82.90XA, S72.91XA, S82.91XA, S72.92XA, S82.92XA, S42.90XA, S52.90XA, S72.90XA, S82.90XA, S22.20XA, S22.49XA, S72.90XA, S82.90XA, S72.91XE, S82.91XB, S72.92XE, S82.92XB, S42.90XB, S52.90XB, S72.90XE, S82.90XB, S22.20XB, S22.49XB, S72.90XE, S82.90XB, T14.8XXA, T14.8XXA, S73.006A, S73.016A, S73.026A, S73.036A, S71.009A, S73.006A, S71.009A, S73.016A, S71.009A, S73.026A, S71.009A, S73.036A, S83.219A, S83.249A, S83.289A, S83.209A, S83.30XA, S83.006A, S81.009A, S83.006A, S83.106A, S83.116A, S83.126A, S83.136A, S83.146A, S83.196A, S81.009A, S83.106A, S81.009A, S83.116A, S81.009A, S83.126A, S81.009A, S83.136A, S81.009A, S83.146A, S81.009A, S83.196A, S93.06XA, S91.009A, S93.06XA, S93.306A, S93.316A, S93.316A, S93.326A, S93.336A, S93.129A, S93.119A, S93.106A, S93.336A, S91.309A, S93.306A, S91.309A, S93.316A, S91.309A, S93.316A, S91.309A, S93.326A, S91.309A, S93.336A, S91.309A, S93.129A, S91.109A, S93.119A, S91.109A, S93.336A, S75.009A, S75.009A, S75.109A, S753.209A, S85.309A, S85.409A, S85.009A, S85.509A, S85.009A, S85.509A, S85.109A, S85.139A, S85.809A, S85.169A, S85.809A, S95.109A, S75.809A,

© 2022 Aday AW et al. *JAMA Network Open.*

Description	ICD-9 Codes	ICD-10 Codes
		S85.809A, S95.809A, S75.909A, S85.909A, S95.902A, T14.90XA, M84.369S, M84.376S, M84.453S, M84.469S, M84.750S, S79.009S, S82.009S, S82.90XS, S92.819S, S92.909S, S92.919S, S99.209S, S99.219S, S99.229S, S99.239S, S99.249S, S99.299S, S77.10XA, S77.00XA, S87.80XA, S87.00XA, S97.80XA, S97.00XA, S97.109A, S77.20XA, S77.20XA, S77.20XA, S77.20XA, S79.819A, S79.829A, S79.919A, S79.929A, S89.80XA, S89.90XA, S99.819A, S99.919A, T84.498A, T84.039A, T84.029A, T84.019A, M97.9XXA, T84.059A, T84.069A, T84.099A, T84.119, T84.129A, T84.199A, T84.498A, T84.50XA, T84.60XA, T84.7XXA, T84.81XA, T84.82XA, T84.83XA, T84.84XA, T84.85XA, T84.86XA, T84.89XA, T84.9XXA

Lower extremity amputations not attributed to peripheral artery disease were identified using these Veterans Health Administration, Veterans Health Administration fee-for-service, or Medicare procedural codes.
ICD, International Classification of Disease; PAD, peripheral artery disease.

© 2022 Aday AW et al. *JAMA Network Open.*

eTable 4. Association of Sex, Race, and Ethnicity With Peripheral Artery Disease Among Individuals Without Diabetes

Characteristic	PAD Events/N	Incidence Rate per 1000 PY [95% CI]	Age-Adjusted, Hazard Ratio [95% CI]	Multivariable-Adjusted Hazard Ratio [95% CI][b]
Sex				
Male	2,086/33,239	14.2 [13.6-14.8]	1.00	1.00
Female	43/2,020	4.2 [3.1-5.7]	0.33 [0.24-0.44]	0.44 [0.32-0.60]
Race/ethnicity[a]				
Black	269/5,373	11.2 [9.9-12.6]	0.83 [0.73-0.95]	0.97 [0.85-1.11]
White	1,623/25,644	14.3 [13.6-15.0]	1.00	1.00
Other	89/1,634	11.9 [9.7-14.7]	0.85 [0.69-1.05]	0.98 [0.79-1.21]
Unknown	148/2,608	12.0 [10.2-14.1]	NA	NA

Qualifying ankle-brachial index studies were restricted to those performed prior to any lower extremity revascularization or amputation.

ABI, ankle-brachial index; CI, confidence interval; PAD, peripheral artery disease; PY, person-years.

a Incidence rates were calculated using observed (i.e., non-imputed) data; missing values were imputed for calculating hazard ratios. Other race and ethnicity included individuals identified as American Indian or Alaska Native, Asian, or Native Hawaiian or other Pacific Islander, as well as those with Hispanic ethnicity.

b Model includes the following independent variables: sex, race/ethnicity, smoking status, age, SBP, DBP, antihypertensive use, BMI, eGFR, HDL-C, LDL-C, triglycerides, statin usage, and prevalent cardiovascular disease. Missing data were multiply-imputed using predictive mean matching.

© 2022 Aday AW et al. *JAMA Network Open.*

eTable 5. Association of Sex, Race, and Ethnicity With Peripheral Artery Disease Among Individual Who Never Smoked

Characteristic	PAD Events/N	Incidence Rate per 1000 PY [95% CI]	Age-Adjusted, Hazard Ratio [95% CI]	Multivariable-Adjusted Hazard Ratio [95% CI][b]
Sex				
Male	853/13,980	12.9 [12.1-13.8]	1.00	1.00
Female	24/992	4.6 [3.1-6.9]	0.38 [0.26-0.58]	0.51 [0.34-0.77]
Race/ethnicity[a]				
Black	188/2,981	13.5 [11.7-15.5]	1.16 [0.98-1.37]	1.18 [0.99-1.40]
White	550/9,742	12.0 [11.0-13.1]	1.00	1.00
Other	81/1,196	13.6 [10.9-16.9]	1.18 [0.95-1.46]	1.05 [0.84-1.31]
Unknown	58/1,053	10.7 [8.3-13.9]	NA	NA

Qualifying ankle-brachial index studies were restricted to those performed prior to any lower extremity revascularization or amputation.

ABI, ankle-brachial index; CI, confidence interval; PAD, peripheral artery disease; PY, person-years.

a Incidence rates were calculated using observed (i.e., non-imputed) data; missing values were imputed for calculating hazard ratios. Other race and ethnicity included individuals identified as American Indian or Alaska Native, Asian, or Native Hawaiian or other Pacific Islander, as well as those with Hispanic ethnicity.

b Model includes the following independent variables: sex, race/ethnicity, diabetes mellitus, age, SBP, DBP, antihypertensive use, BMI, eGFR, HDL-C, LDL-C, triglycerides, statin usage, and prevalent cardiovascular disease. Missing data were multiply-imputed using predictive mean matching.

© 2022 Aday AW et al. *JAMA Network Open.*

eTable 6. Association of Sex, Race, and Ethnicity With Peripheral Artery Disease Among Individuals Without Diabetes Who Never Smoked

Characteristic	PAD Events/N	Incidence Rate per 1000 PY [95% CI]	Age-Adjusted, Hazard Ratio [95% CI]	Multivariable-Adjusted Hazard Ratio [95% CI][c]
Sex				
Male	128/5,481	5.3 [4.4-6.3]	1.00	1.00
Female	<10/632[b]	2.1 [1.0-4.5]	0.50 [0.23-1.08]	0.58 [0.26-1.30]
Race[a]				
Black	20/1,084	4.2 [2.7-6.4]	1.02 [0.66-1.58]	1.14 [0.73-1.79]
White	93/4,155	5.0 [4.1-6.2]	1.00	1.00
Other	<10/404[b]	4.3 [2.2-8.7]	1.22 [0.67-2.22]	1.21 [0.66-2.19]
Unknown	14/470	5.8 [3.4-9.7]	NA	NA

Qualifying ankle-brachial index studies were restricted to those performed prior to any lower extremity revascularization or amputation.

ABI, ankle-brachial index; CI, confidence interval; PAD, peripheral artery disease; PY, person-years.

a Incidence rates were calculated using observed (i.e., non-imputed) data; missing values were imputed for calculating hazard ratios. Other race and ethnicity included individuals identified as American Indian or Alaska Native, Asian, or Native Hawaiian or other Pacific Islander, as well as those with Hispanic ethnicity.

b Privacy rules for Medicare data require counts less than 10 be reported as "<10" rather than discrete counts.

c Model includes the following independent variables: sex, race/ethnicity, age, SBP, DBP, antihypertensive use, BMI, eGFR, HDL-C, LDL-C, triglycerides, statin usage, and prevalent cardiovascular disease. Missing data were multiply-imputed using predictive mean matching.

© 2022 Aday AW et al. *JAMA Network Open.*

eTable 7. Baseline Characteristics of Individuals Without Diabetes Who Never Smoked

	Patients, No. (%)				
**Characteristic**	**Men (n = 5,481)[a]**	**Women (n = 632)[b]**	**Black (n = 1,084)[c]**	**White (n = 4,155)[d]**	**Other (n = 404)[e]**
Age, mean (SD), y	60.4 (6.5)	55.5 (6.8)	57.4 (6.7)	60.7 (6.4)	59.0 (6.7)
Male	NA	NA	906 (83.6)	3,789 (91.2)	372 (92.1)
Race/ethnicity					
Black	906 (17.9)	178 (30.9)	NA	NA	NA
White	3,789 (74.8)	366 (63.5)	NA	NA	NA
Other[f]	372 (7.3)	32 (5.6)	NA	NA	NA
Prevalent CVD	1,689 (30.8)	92 (14.6)	290 (26.8)	1,270 (30.6)	107 (26.5)
Left ankle-brachial index, median (Q1, Q3)	1.1 (1.0, 1.2)	1.1 (1.0, 1.2)	1.1 (1.0, 1.2)	1.1 (1.0, 1.2)	1.1 (1.0, 1.2)
Right ankle-brachial index, median (Q1, Q3)	1.1 (1.0, 1.2)	1.1 (1.0, 1.2)	1.1 (1.0, 1.2)	1.1 (1.0, 1.2)	1.1 (1.0, 1.2)
SBP, mean (SD), mmHg	128.8 (12.3)	126.3 (13.6)	129.4 (12.9)	128.3 (12.2)	128.5 (12.1)
DBP, mean (SD), mmHg	77.8 (8.1)	75.7 (8.2)	78.9 (8.5)	77.2 (8.0)	78.1 (8.3)
Antihypertensive therapy	4,994 (91.1)	527 (83.4)	994 (91.7)	3,772 (90.8)	366 (90.6)
BMI, mean (SD), kg/m^2	31.2 (6.5)	30.7 (7.2)	31.0 (6.6)	31.2 (6.6)	30.7 (5.7)
Total cholesterol, median (Q1, Q3), mg/dL	177 (153, 204)	195 (171, 222)	179 (157, 205)	177 (153, 206)	185 (158, 211)
LDL cholesterol, median (Q1, Q3), mg/dL	105 (83, 129)	115 (92, 137)	1066 (86, 132)	105 (83, 129)	108 (86, 134)
HDL cholesterol, median (Q1, Q3), mg/dL	43 (36, 52)	55 (46, 67)	48 (40, 58)	43 (36, 52)	43 (35, 52)
Triglycerides, median (Q1, Q3), mg/dL	119 (83, 175)	106 (73, 148)	93 (68, 138)	123 (86, 179)	132 (93, 196)
Statin therapy	3,732 (68.1)	340 (53.8)	679 (62.6)	2,847 (68.5)	274 (67.8)
eGFR, mean (SD), mL/min/1.73m^2	86.4 (24.4)	84.7 (20.7)	90.3 (27.6)	85.3 (23.1)	86.9 (21.9)

SI conversion factors: To convert LDL and HDL cholesterol to mmol/L, multiply values by 0.0259. To convert triglycerides to mmol/L, multiply by 0.0113.

BMI, body-mass index; CVD, cardiovascular disease; DBP, diastolic blood pressure; eGFR, estimated glomerular filtration rate; HDL, high-density lipoprotein; LDL, low-density lipoprotein; PAD, peripheral artery disease; SBP, systolic blood pressure; SD, standard deviation.

a Number missing: 414 (Race/ethnicity); 131 (SBP); 131 (DBP); 61 (BMI); 166 (Total cholesterol); 208 (LDL cholesterol); 200 (HDL cholesterol); 206 (Triglycerides); 81 (eGFR)

b Number missing: 56 (Race/ethnicity); 64 (SBP); 64 (DBP); 13 (BMI); 21 (Total cholesterol); 28 (LDL cholesterol); 27 (HDL cholesterol); 25 (Triglycerides); 15 (eGFR)

c Number missing: 35 (SBP); 35 (DBP); 9 (BMI); 31 (Total Cholesterol); 47 (LDL Cholesterol); 41 (HDL Cholesterol); 37 (Triglycerides); 9 (eGFR)

d Number missing: 113 (SBP); 113 (DBP); 40 (BMI); 108 (Total Cholesterol); 128 (LDL Cholesterol); 125 (HDL Cholesterol); 132 (Triglycerides); 60 (eGFR)

e Number missing: 13 (SBP); 13 (DBP); 7 (BMI); 19 (Total Cholesterol); 20 (LDL Cholesterol); 20 (HDL Cholesterol); 21 (Triglycerides); 7 (eGFR)

f Other race and ethnicity included individuals identified as American Indian or Alaska Native, Asian, or Native Hawaiian or other Pacific Islander, as well as those with Hispanic ethnicity.

© 2022 Aday AW et al. *JAMA Network Open.*

eTable 8. Deaths During Follow-up by Sex, Race and Ethnicity, Diabetes, and Smoking

Characteristic	Deaths/N (%)
Sex	
Male	10,870/73,870 (14.7)
Female	208/3,219 (6.5)
Race/ethnicity	
Black	1,659/13,080 (12.7)
White	7,543/54,377 (13.9)
Other[a]	532/4,454 (11.9)
Diabetes Mellitus	
Absent	4,412/35,259 (12.5)
Present	6,666/41,782 (16.0)
Smoking Status	
Never	1,523/14,972 (10.2)
Former	2,271/18,348 (12.4)
Current	5,237/29,263 (17.9)

a Other race and ethnicity included individuals identified as American Indian or Alaska Native, Asian, or Native Hawaiian or other Pacific Islander, as well as those with Hispanic ethnicity.

© 2022 Aday AW et al. *JAMA Network Open.*

eTable 9. Association of Sex, Race and Ethnicity, Diabetes, and Smoking With Subsequent Ankle-Brachial Index Less Than 0.90

Characteristic	PAD Events/N	Incidence Rate per 1000 PY [95% CI]	Age-Adjusted, Hazard Ratio [95% CI]	Multivariable-Adjusted Hazard Ratio [95% CI][a]
Sex				
Male	4,454/73,822	12.7 [12.4-13.1]	1.00	1.00
Female	82/3,219	4.9 [4.0-6.1]	0.42 [0.34-0.53]	0.54 [0.43-0.67]
Race/ethnicity[b,c]				
Black	737/13,080	11.8 [11.0-12.7]	0.96 [0.88-1.04]	1.04 [0.95-1.13]
White	3,327/54,377	12.9 [12.5-13.4]	1.00	1.00
Other	225/4,454	10.2 [9.0-11.6]	0.80 [0.70-0.92]	0.86 [0.75-0.99]
Unknown	247/5,130	10.0 [8.8-11.3]	NA	NA
Diabetes Mellitus				
Absent	1,683/35,259	10.6 [10.1-11.1]	1.00	1.00
Present	2,853/41,782	13.7 [13.3-14.3]	1.29 [1.21-1.37]	1.31 [1.22-1.40]
Smoking Status[b]				
Never	448/14,972	6.2 [5.6-6.8]	1.00	1.00
Former	899/18,348	10.9 [10.2-11.6]	1.59 [1.42-1.78]	1.47 [1.31-1.64]
Current	2,448/29,263	17.1 [16.5-17.8]	2.68 [2.42-2.95]	2.27 [2.05-2.51]
Unknown	741/14,458	10.8 [10.1-11.7]	NA	NA

Qualifying ankle-brachial index studies were restricted to those performed prior to any lower extremity revascularization or amputation.

ABI, ankle-brachial index; CI, confidence interval; PAD, peripheral artery disease; PY, person-years.

a Model includes the following independent variables: sex, race/ethnicity, diabetes mellitus, smoking status, age, SBP, DBP, antihypertensive use, BMI, eGFR, HDL-C, LDL-C, triglycerides, statin usage, and prevalent cardiovascular disease. Missing data were multiply-imputed using predictive mean matching.

b Incidence rates were calculated using observed (i.e., non-imputed) data; missing values were imputed for calculating hazard ratios.

c Other race and ethnicity included individuals identified as American Indian or Alaska Native, Asian, or Native Hawaiian or other Pacific Islander, as well as those with Hispanic ethnicity.

© 2022 Aday AW et al. *JAMA Network Open.*

eFigure 1. Sample Derivation

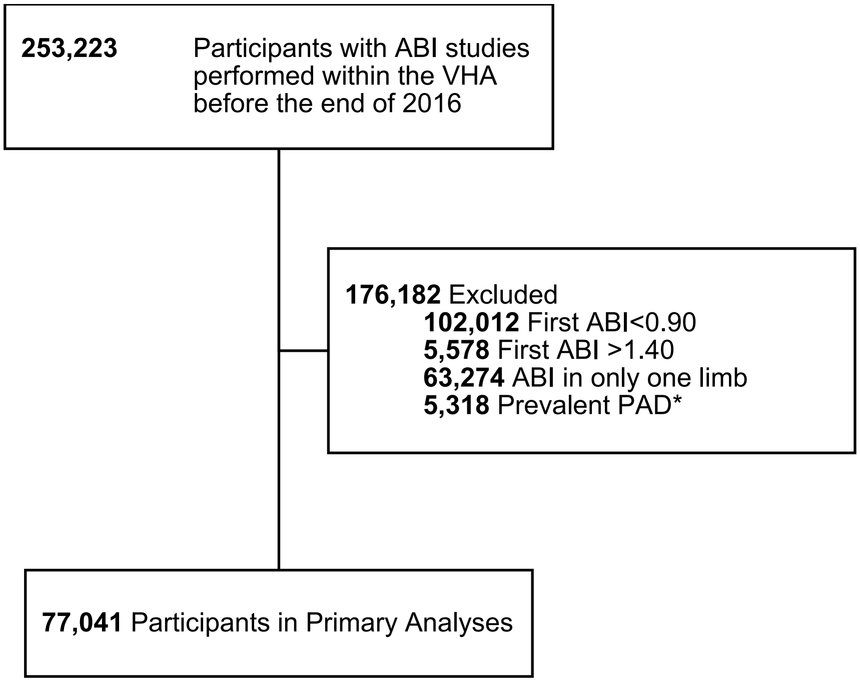

The study population was selected from individuals with ankle-brachial index studies performed within the Veterans Health Administration healthcare system without prevalent peripheral artery disease at baseline.
ABI, ankle-brachial index; PAD, peripheral artery disease; VHA, Veterans Health Administration.
*Based on International Classification of Disease-9/10 and current procedural terminology codes

© 2022 Aday AW et al. *JAMA Network Open.*

eFigure 2. Cumulative Incidence of Peripheral Artery Disease by Smoking History

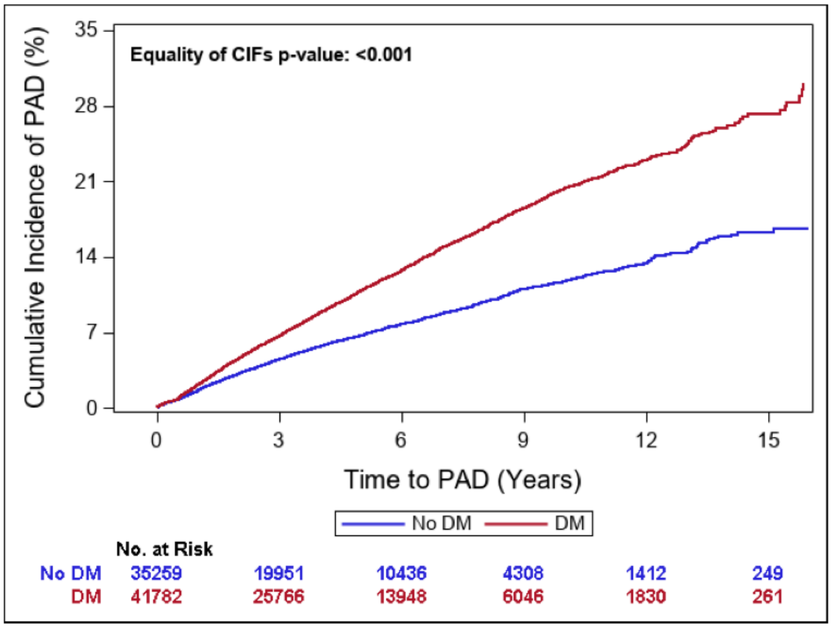

Cumulative incidence of peripheral artery disease stratified by history of diabetes mellitus. Incident peripheral artery disease was defined by ankle-brachial index <0.90, non-traumatic amputation, or surgical or percutaneous lower extremity arterial revascularization.
CIF, cumulative incidence function.

© 2022 Aday AW et al. *JAMA Network Open.*

eFigure 3. Cumulative Incidence of Peripheral Artery Disease by Smoking History

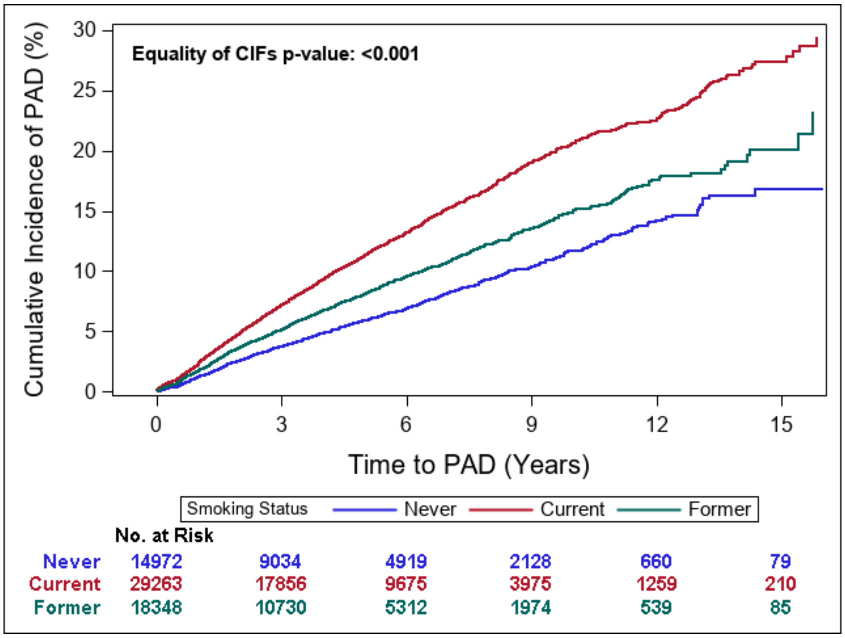

Cumulative incidence of peripheral artery disease stratified by smoking history. Incident peripheral artery disease was defined by ankle-brachial index <0.90, non-traumatic amputation, or surgical or percutaneous lower extremity arterial revascularization.
CIF, cumulative incidence function.

© 2022 Aday AW et al. *JAMA Network Open.*

eFigure 4. Distribution of Time to Incident Peripheral Artery Disease by Race

Histogram depicting distribution of time from baseline to **(A)** overall incident peripheral artery disease, **(B)** lower extremity revascularization, **(C)** non-traumatic lower extremity amputation, and **(D)** subsequent ankle-brachial index <0.90 stratified by race.

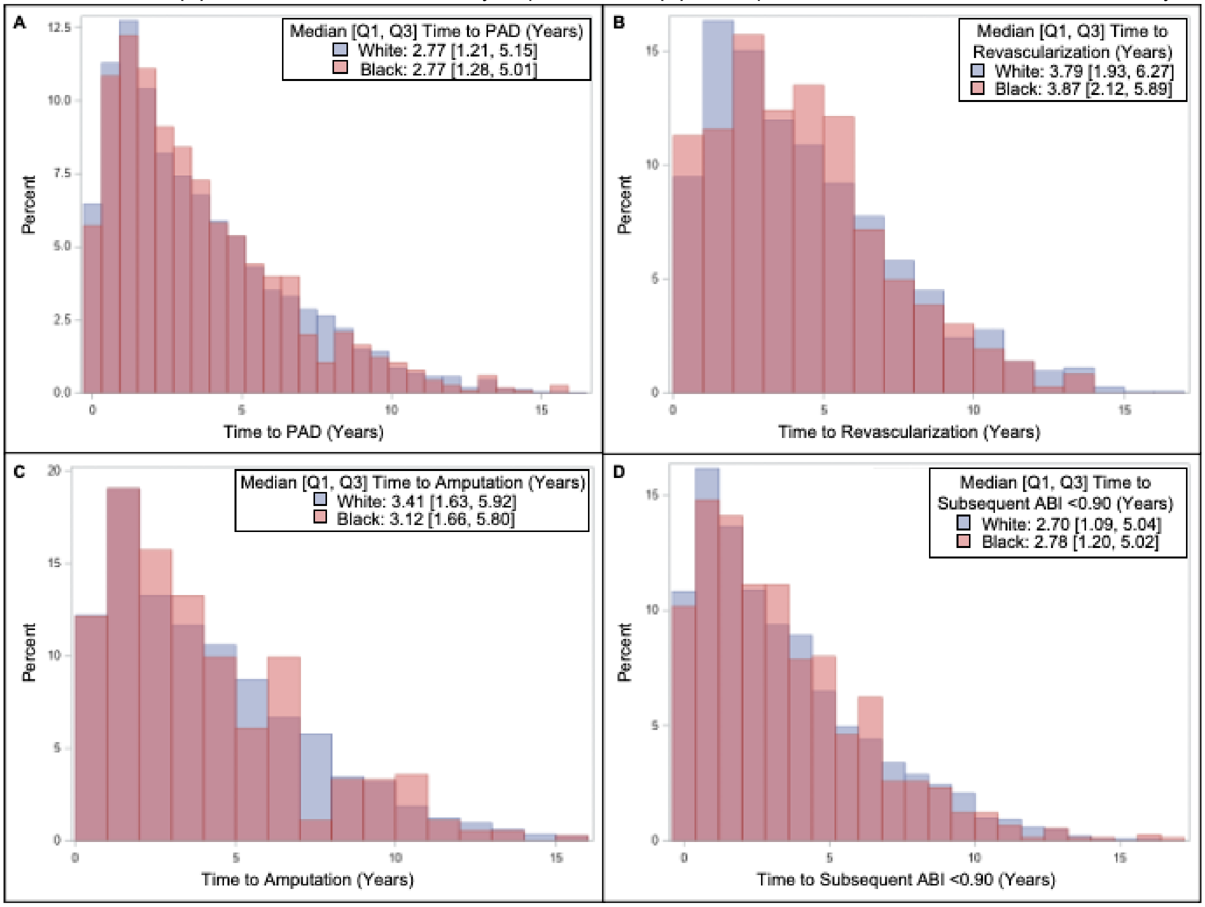

The legend also includes median and interquartile range of time to each endpoint. Analyses are limited to individuals who developed each incident outcome.

© 2022 Aday AW et al. *JAMA Network Open.*
